# Construction of relatedness matrices in autopolyploid populations using low-depth high-throughput sequencing data

**DOI:** 10.1007/s00122-024-04568-2

**Published:** 2024-03-02

**Authors:** Timothy P. Bilton, Sanjeev Kumar Sharma, Matthew R. Schofield, Michael A. Black, Jeanne M. E. Jacobs, Glenn J. Bryan, Ken G. Dodds

**Affiliations:** 1 AgResearch, Invermay Agricultural Centre, Mosgiel, New Zealand; 2 Department of Mathematics and Statistics, University of Otago, Dunedin, New Zealand; 3 Cell and Molecular Sciences, The James Hutton Institute, Invergowrie, Dundee, UK; 4 Department of Biochemistry, University of Otago, Dunedin, New Zealand; 5 AgResearch, Lincoln Science Centre, Christchurch, New Zealand

## Abstract

**Key message:**

An improved estimator of genomic relatedness using low-depth high-throughput sequencing data for autopolyploids is developed. Its outputs strongly correlate with SNP array-based estimates and are available in the package GUSrelate.

**Abstract:**

High-throughput sequencing (HTS) methods have reduced sequencing costs and resources compared to array-based tools, facilitating the investigation of many non-model polyploid species. One important quantity that can be computed from HTS data is the genetic relatedness between all individuals in a population. However, HTS data are often messy, with multiple sources of errors (i.e. sequencing errors or missing parental alleles) which, if not accounted for, can lead to bias in genomic relatedness estimates. We derive a new estimator for constructing a genomic relationship matrix (GRM) from HTS data for autopolyploid species that accounts for errors associated with low sequencing depths, implemented in the *R* package GUSrelate. Simulations revealed that GUSrelate performed similarly to existing GRM methods at high depth but reduced bias in self-relatedness estimates when the sequencing depth was low. Using a panel consisting of 351 tetraploid potato genotypes, we found that GUSrelate produced GRMs from genotyping-by-sequencing (GBS) data that were highly correlated with a GRM computed from SNP array data, and less biased than existing methods when benchmarking against the array-based GRM estimates. GUSrelate provides researchers with a tool to reliably construct GRMs from low-depth HTS data.

**Supplementary Information:**

The online version contains supplementary material available at 10.1007/s00122-024-04568-2.

## Introduction

Genetic relatedness information is central to several types of analyses in quantitative and conservation genetics. In breeding applications, estimation of genetic variance components, which are used in the estimation of breeding values and genomic selection applications, rely on relatedness information (Falconer 1981; Lynch and Walsh 1998). Conservation management programs utilize relatedness information to inform mating decisions that seek to avoid inbreeding depression in wild populations while increasing genetic variability (Oliehoek et al. 2006; Wang 2004). Both breeding and conservation programs require pedigree information for mating allocations and incomplete pedigrees or pedigree errors are frequently encountered in practice (Oliehoek and Bijma 2009). Relatedness values can also be used to perform quality control of recorded pedigrees and to assign parentage on individuals missing in a pedigree (Bradford et al. 2019; Dodds et al. 2019; Munoz et al. 2014).

Genetic relatedness is typically described in terms of a concept known as identity-by-descent (IBD), defined as the event that two alleles are copies of an allele from a common ancestor. A plethora of methods and software for estimating relatedness have been developed for diploid species (Weir et al. 2006). The simplest and earliest approach was to use a pedigree (Henderson 1976; Quaas 1976), but this relies on complete and accurate pedigree records which are not always available in practice. Later methods utilizing marker data to estimate relatedness were developed, and fall under two classes of estimators; method-of-moments (Li et al. 1993; Lynch and Ritland 1999; Queller and Goodnight 1989; Wang 2002) and maximum likelihood (Anderson and Weir 2007; Choi et al. 2009; Milligan 2003; Thompson 1975). These methods determine relatedness by estimating the probabilities of various IBD configurations. An alternative to estimating IBD probabilities is to estimate the proportion of genome with shared ancestry directly in the form of a relationship matrix, which is a symmetric matrix specifying the relationship between an individual with all other individuals in a population, including itself. A relationship matrix constructed using genetic marker data is known as a genomic relationship matrix (GRM) and provides a method-of-moments estimator of genetic relatedness.

Estimation of relatedness in polyploids is more complicated than in diploids. For example, polysomic inheritance results in more complex IBD configurations (Huang et al. 2015a). Polyploid species are generally classified into allopolyploids or autopolyploids. In allopolyploids, disomic inheritance is generally assumed so that relatedness estimation can be carried out under a diploid framework, which is not the case for autopolyploids, as polysomic inheritance is typically present (Bourke et al. 2018). The development of methods for analysing autopolyploids that account for the peculiar inheritance patterns found in these species has lagged behind the development of methods for diploid species (Amadeu et al. 2020). Nevertheless, within the last decade, several new relatedness estimators have been developed for autopolyploid species. All these methods are extensions of estimators developed for diploid species and include the pedigree numerator relationship matrix (Hamilton and Kerr 2018; Kerr et al. 2012; Slater et al. 2014), several method-of-moments estimators (Huang et al. 2015b, 2014), a maximum likelihood estimator (Huang et al. 2015a, 2015b) and various extensions of the VanRaden (2008) (method 1) GRM estimator (Amadeu et al. 2016; Ashraf et al. 2016; Batista et al. 2022; Cericola et al. 2018; de Bem Oliveira et al. 2019; Pembleton et al. 2013; Slater et al. 2016; Zingaretti et al. 2019). Endelman et al. (2018), in particular, derived the VanRaden (2008) (method 1) estimator for autotetraploids from first principles. Amadeu et al. (2020) recently conducted a study comparing the performance of a range of these estimators. Their recommendation was the VanRaden type GRM method with reliable dosage information as the best approach to use, provided there were more than 100 SNPs available.

The original relatedness estimators were developed in conjunction with the early genetic marker technologies, such as restriction fragment length polymorphism markers and simple sequence repeat (multiallelic) markers, that were costly, low throughput and only generated at most a few hundred markers. The development of SNP array technologies improved throughput and generated considerably more (biallelic) markers enabling better estimation of genetic relatedness and thereby improving the power of many genetic studies. In recent times, the introduction of reduced representation high-throughput sequencing (HTS) techniques, such as whole-exome sequencing (Hodges et al. 2007), restriction-site associated DNA sequencing (Baird et al. 2008), and genotyping-by-sequencing (GBS) (Dodds et al. 2015; Elshire et al. 2011), has further reduced the cost and time resources required for obtaining large numbers of markers. Application of HTS methods on autopolyploids have increased in popularity in recent times and have been applied on a range of species including alfalfa (*Medicago sativa* L.) (Munjal et al. 2018), blueberry (*Vaccinium corymbosum*) (de Bem Oliveira et al. 2019; McCallum et al. 2016), potato (*Solanum tuberosum* L.) (Sverrisdottir et al. 2017; Uitdewilligen et al. 2013), seashore paspalum (*Paspalum vaginatum*) (Goad et al. 2021), sugarcane (*Saccharum* spp.) (Wang et al. 2022), sweetpotato (*Ipomoea batatas*) (Shirasawa et al. 2017), and Urochloa spp. (Matias et al. 2019).

A major issue, however, with HTS technology is that the data generated is messy and contains multiple sources of error (Gerard et al. 2018). One type of error prevalent in HTS data is unobserved alleles (also sometimes referred to as missing parental alleles in diploids), which is when one or more of an individual’s alleles have not been observed at a locus due to low read depths. If this error is not accounted for it can result in heterozygous genotypes being inferred as homozygous or the wrong dosage to be called for heterozygous genotypes. Dodds et al. (2015) and Ashraf et al. (2016) have shown that for diploids, missing parental alleles in HTS data result in a considerable upward bias on self-relatedness (i.e. inbreeding) estimates but have no impact on the bias of relatedness (i.e. coancestry) estimates between pairs of individuals, provided missing genotypes are correctly accounted for. Missing parental alleles can also lead to no reads being observed at a locus for a given individual, which results in a missing genotype. This can further complicate relatedness estimation as some methods require complete genotype calls, while imputing genotypes can lead to bias in relatedness estimates. The problem of missing parental alleles is exacerbated as the average read depth decreases. Another type of error present in HTS data is sequencing error, which is where a nucleotide base has been called incorrectly during sequencing (Bilton et al. 2018a, 2018b; Li 2011). Sequencing errors will result in incorrect genotype calls if not accounted for, but the impact of these errors on relatedness estimation in the context HTS data has not been examined.

Methods for estimating relatedness using HTS data have been presented in the literature. Ackerman et al. (2017), Hanghoj et al. (2019) and Korneliussen and Moltke (2015) extended several maximum likelihood estimators for genotypic data from low-coverage sequencing data to account for the uncertainty associated with the genotype calls. Dodds et al. (2015) derived an extension of the VanRaden (2008) (method 1) GRM to HTS data that accounts for missing parental alleles due to low read depths. However, all these HTS relatedness estimators are only applicable to diploid species. One exception is the estimator developed by Cericola et al. (2018), which extends the VanRaden (2008) (method 1) GRM to pooled HTS data, but it is yet to be tested in autopolyploid populations. Alternatively, one could estimate relatedness from HTS data by first performing genotype calling using appropriate software, e.g. EBG (Blischak et al. 2018), updog (Gerard et al. 2018), polyRAD (Clark et al. 2019), and then using one of the relatedness estimators developed for autopolyploids. The issue with this approach is that accurately inferring genotypes from low read depth HTS data is difficult, and errors in genotype calls are likely to be present unless the read depth is sufficiently high. One way to mitigate this problem is to sequence at a higher average read depth and discard genotype calls with read depths below some threshold value. However, for a given sequencing cost, this requires sequencing fewer individuals and SNPs, and discarding a considerable amount of the data generated may not be an optimal use of resources (Dodds et al. 2015). To date, the most promising approach for constructing GRMs in autopolyploids using HTS data has been to use the ratio of reference reads to the total number of reads as a proxy for the genotype call (de Bem Oliveira et al. 2019), but this still has the problem that self-relatedness estimates will be inflated if the read depths are low.

In this article, we provide a mathematical justification of the mixed-ploidy autopolyploid GRM estimator found in the literature that is based on the VanRaden (2008) (method 1) estimator. Based on this estimator, we then develop a new GRM estimator for autopolyploid species using HTS data that accounts for missing parental alleles and sequencing errors present in the data. Our new GRM estimator for HTS data can be considered an extension of the estimator developed by Dodds et al. (2015) to autopolyploid species and to account for sequencing errors. The performance of our estimator is tested and compared with some existing approaches for constructing GRMs on autopolyploids using simulated data and a real HTS dataset.

## Material and methods

Denote the coancestry coefficient by ${\theta }_{hi}$ which is the probability that two random alleles between individuals $h$ and $i$ are identical-by-descent (IBD), where $h,i=1,\dots ,N$ and $N$ is the total number of individuals in the population. When $h=i$, the coancestry coefficient is ${\theta }_{ii}=(1+{({\psi }_{i}-1)F}_{i})/{\psi }_{i}$(Gallais 2003, Eq. 3.6a), where ${F}_{i}$ denotes the inbreeding coefficient which is the probability that two alleles from individual $i$ are IBD, and ${\psi }_{i}$ denotes the ploidy level of individual $i$ (${\psi }_{i}\in \{2, 3,\dots \})$. A measure of genetic relatedness between individuals $i$ and $j$ is given by the relatedness coefficient defined as ${r}_{hi}=\sqrt{{\psi }_{h}{\psi }_{i}}{\theta }_{hi}$ (Hamilton and Kerr 2018). When $h=i$, the coefficient ${r}_{ii}={\psi }_{i}{\theta }_{ii}=1+{({\psi }_{i}-1)F}_{i}$ is known as the self-relatedness coefficient. In the next section, we derive an estimator of ${r}_{hi}$ for autopolyploids based on the VanRaden (2008) GRM estimator for genotypic and HTS data.

### Relatedness estimators for autopolyploids

#### Genotypic data

Let ${X}_{ij}$ denote the number of major alleles from individual $i$ at SNP $j$, where $j=1,\dots ,M$ and $M$ is the number of SNPs. VanRaden (2008) (method 1) proposed the GRM estimator for estimating relatedness between individuals $h$ and $i$ using genotypic data in diploid populations as

1 $${G}_{hi}^{{\text{VR}}}=\frac{{\sum }_{j=1}^{M}({X}_{hj}-2{p}_{j})({X}_{ij}-2{p}_{j}) }{2{\sum }_{j=1}^{M}{p}_{j}(1-{p}_{j})},$$

where ${p}_{j}$ is the ancestral major allele frequency at SNP $j$ for individuals in the reference population. In practice, the ancestral allele frequencies are rarely known and typically estimated using the sample allele frequencies from the population, that is, ${p}_{j}$ is replaced by

2 $${\widehat{p}}_{j}=\frac{1}{2N}{\sum }_{i=1}^{N}{X}_{ij}.$$

When ${p}_{j}$ is known, the expected value of ${G}_{hi}^{{\text{VR}}}$ when $h\ne i$ is equal to the relatedness, $E\left({G}_{hi}^{{\text{VR}}}\right)=2{\theta }_{hi}$, while the expected value of ${G}_{hi}^{{\text{VR}}}$ when $h=i$ is equal to the self-relatedness coefficient, $E\left({G}_{ii}^{{\text{VR}}}\right)=1+{F}_{i}$. Substituting the sample allele frequency estimates from Eq. (2) for ${p}_{j}$ introduces bias into the GRM estimator, although if the population is relatively unrelated overall, this bias is small (Wang 2014).

We now show the derivation of the autopolyploid version of the VanRaden estimator given in Eq. (1). In autopolyploids, it is typically assumed that the pairing of homologous chromosomes during meiosis occurs randomly (i.e. assuming HWE), regardless of whether pairing occurs in bivalent or multivalent formation (Gallais 2003). Under this assumption, the alleles an individual inherits from a parent are a random sample of the alleles found in that parent. It follows that the alleles an individual inherits are a random sample from the pool of alleles found in the reference population, and thus a plausible genotype model under HWE for autopolyploids is the binomial model ${\text{Bin}}({\psi }_{i},{p}_{j})$, where ${\text{Bin}}(n,p)$ denotes a binomial distribution with $n$ trials and probability of success $p$. This assumes that the alleles sampled within a parent are independent, which may not be the case, as alleles can be IBD due to inbreeding. One form of inbreeding that is not present in diploid or allopolyploid species is double reduction, which is where gametes produced by meiosis have segments that are derived from the same parental chromosome which can only occur when there is multivalent pairing (Gallais 2003). Modelling genotype probabilities in the presence of inbreeding using IBD probabilities is difficult in GRMs. This problem is not unique to polyploids and has been discussed for diploids where an alternative approach is to model inbreeding as a correlation of alleles instead of IBD probabilities (Powell et al. 2010). Therefore, an alternate genotype model for autopolyploids is to model the coancestry coefficient ${\theta }_{hi}$ as the correlation of two alleles between individuals and the inbreeding coefficient ${F}_{i}$ as the correlation of two alleles within an individual. This leads to using a correlated binomial model and it follows that (Ahn and Chen 1995)

3 $$E\left({X}_{ij}\right)={\psi }_{i}{p}_{j}$$

4 $$E\left({X}_{ij}^{2}\right)={\psi }_{i}{p}_{j}\left(1-{p}_{j}\right)\left(1+\left({\psi }_{i}-1\right){F}_{i}\right)+ {\psi }_{i}^{2}{p}_{j}^{2}$$

5 $$E\left({X}_{hj}{X}_{ij}\right)={\psi }_{h}{\psi }_{i}\left({p}_{j}\left(1-{p}_{j}\right){\theta }_{hi}+{p}_{j}^{2}\right).$$

Note that Eq. (4) is equivalent to Eq. (5) when $h=i$. If we set the expectation $E\left[\left({X}_{hj}-{\psi }_{h}{p}_{j}\right)\left({X}_{ij}-{\psi }_{i}{p}_{j}\right)\right]$ to its sample quantity, then we use Eqs. (3)-(5) to derive the following method-of-moments GRM estimator for a mixed-ploidy autopolyploid population,

6 $${G}_{hi}^{{\text{VRP}}}=\frac{{\sum }_{j=1}^{M}({X}_{hj}-{\psi }_{h}{p}_{j})({X}_{ij}-{\psi }_{i}{p}_{j}) }{\sqrt{{\psi }_{h}{\psi }_{i}}{\sum }_{j=1}^{M}{p}_{j}(1-{p}_{j})}.$$

Full derivations of Eq. (6) are given in Supplementary File 1. As in the diploid case, when the ancestral allele frequencies are known, the expected value of the estimator in Eq. (6) is equal to the relatedness coefficient when $h\ne i$, that is $E\left({G}_{hi}^{{\text{VRP}}}\right)=\sqrt{{\psi }_{h}{\psi }_{i}}{\theta }_{hi}$, while the expected value of ${G}_{hi}^{{\text{VRP}}}$ is equal to the self-relatedness coefficient when $h=i$, that is $E\left({G}_{ii}^{{\text{VRP}}}\right)=1+{({\psi }_{i}-1)F}_{i}$. Similar to Eq. (2), the ancestral allele frequencies are estimated via

7 $${\widehat{p}}_{j}=\frac{1}{N}{\sum }_{i=1}^{N}\frac{{X}_{ij}}{{\psi }_{i}}.$$

The GRM estimator in Eq. (6) has been used in the literature, mainly for tetraploid populations when ${\psi }_{i}=4$ (Ashraf et al. 2016; Zingaretti et al. 2019) but also for mixed ploidy species (Batista et al. 2022). Our contribution is to justify this estimator mathematically for mixed-ploidy populations. Note that Eq. (6) can be re-expressed in the simpler form of

8 $${G}_{hi}^{{\text{VRP}}}=\sqrt{{\psi }_{h}{\psi }_{i}}\frac{{\sum }_{j=1}^{M}({R}_{hj}-{p}_{j})({R}_{ij}-{p}_{j}) }{{\sum }_{j=1}^{M}{p}_{j}(1-{p}_{j})},$$

where ${R}_{ij}={X}_{ij}/{\psi }_{i}$ is the proportion of reference alleles in the genotype. Equation (8) highlights that the practice of scaling genotype calls into a diploid framework in autopolyploids, which has been previously used (Pembleton et al. 2013) and results in the same estimates as in Eq. (6).

#### Sequencing data

For HTS data, genotype information is unobserved (i.e. latent) and the errors in the data make accurately inferring genotypes difficult. The observed data are the number of reads for the reference and alternate alleles. Let ${Y}_{ij}$ denote the number of observed reads for the reference allele in individual $i$ at SNP $j$, where ${Y}_{ij}$ is an integer value between 0 and ${d}_{ij}$, and ${d}_{ij}$ is the sequencing (read) depth in individual $i$ at SNP $j$. A common approach for modelling HTS data is to assume that ${Y}_{ij}$ arises from a binomial sample of the alleles in the genotype ${X}_{ij}$ with a constant rate of sequencing errors between reads (Bilton et al. 2018b; Blischak et al. 2016; Dodds et al. 2015; Li 2011). Thus,

9 $$P\left({Y}_{ij}|{X}_{ij}={x}_{ij}\right)\sim {\text{Bin}}({d}_{ij},{\pi }_{ij}),$$

where

10 $${\pi }_{ij}=\frac{{x}_{ij}}{{\psi }_{i}}\left(1-{\varepsilon }_{j}\right)+\left(1-\frac{{x}_{ij}}{{\psi }_{i}}\right){\varepsilon }_{j},$$

and ${\varepsilon }_{j}$ is the sequencing error rate (i.e. the probability of a base being incorrectly called during sequencing) at SNP $j$ and ${x}_{ij}$ is unobserved. The quantity ${\pi }_{ij}$ denotes the probability of observing a reference allele in the HTS dataset from individual $i$ at SNP $j$. A reference allele is observed when either (1) a reference allele from the genotype is sequenced without error (with probability ${x}_{ij}(1-{\varepsilon }_{j})/{\psi }_{i}$) or (2) the alternate allele is sequenced with error (with probability $(1-{x}_{ij}/{\psi }_{i}){\varepsilon }_{j}$).

One approach for estimating relatedness in polyploids with HTS data has been to use the VanRaden estimator with the ratio of the number of reference reads to the total number of reads used as a proxy for the genotype call (de Bem Oliveira et al. 2019; Sverrisdottir et al. 2017). This equates to replacing ${X}_{ij}$ with ${Z}_{ij}={\psi }_{i}{Y}_{ij}/{d}_{ij}$ in Eq. (6). This approach is valid provided that the read depth for each SNP is sufficiently high so that errors resulting from low read depths are minimal. However, low read depths in the data can result in underestimation of self-relatedness using the ratio approach (Ashraf et al. 2016; Dodds et al. 2015). We now derive an adjustment to the VanRaden estimator for HTS data that accounts for errors in the data and uses ${Z}_{ij}$ for the genotype call.

Using the binomial model specified in Eq. (9), it follows from the law of total probability that

11 $$E\left({Z}_{ij}\right)={\psi }_{i}{\varepsilon }_{j}+\left(1-2{\varepsilon }_{j}\right)E\left({X}_{ij}\right)$$

12 $$E\left({Z}_{ij}^{2}\right)={\psi }_{i}^{2}{\varepsilon }_{j}\left(1-\left(1-{\varepsilon }_{j}\right){\delta }_{ij}\right)+{\psi }_{i}\left(1-2{\varepsilon }_{j}-{\gamma }_{j}{\delta }_{ij}\right)E({X}_{ij})+{\gamma }_{j}{\delta }_{ij}E({X}_{ij}^{2})$$

13 $$E\left({Z}_{hj}{Z}_{ij}\right)={\gamma }_{j}E\left({X}_{hj}{X}_{ij}\right)+{\varepsilon }_{j}\left(1-2{\varepsilon }_{j}\right)\left({\psi }_{i}E\left({X}_{hj}\right)+{\psi }_{h}E\left({X}_{ij}\right)\right)+{\psi }_{h}{\psi }_{i}{\varepsilon }_{j}^{2}$$

where ${\gamma }_{j}=1-4{\varepsilon }_{j}\left(1-{\varepsilon }_{j}\right)$ and ${\delta }_{ij}=1-1/{d}_{ij}$. See Supplementary File 1 for derivations of Eqs. (11)–(13). When there is no sequencing error (${\varepsilon }_{j}=0$), Eq. (13) simplifies to $E\left({X}_{hj}{X}_{ij}\right)$ which means that estimating relatedness between two individuals is not affected by errors due to low read depths. This is consistent with the diploid case (Dodds et al. 2015) and was shown to occur in the polyploid case via simulations (Ashraf et al. 2016).

If we set the expectation $E\left[\left({Z}_{hj}-{\psi }_{h}{p}_{j}\right)\left({Z}_{ij}-{\psi }_{i}{p}_{j}\right)\right]$ to its sample quantity, then from Eqs. (11)–(13) we can derive the following method-of-moments GRM estimator for a mixed-ploidy autopolyploid population with HTS data as

14 $${G}_{hi}=\sqrt{{\psi }_{h}{\psi }_{i}}\frac{{\sum }_{j=1}^{M}\left[\left({S}_{hj}-{p}_{j}\right)\left({S}_{ij}-{p}_{j}\right)-{\varepsilon }_{j}^{2}{\eta }_{j}\right]/{\gamma }_{j}}{{\sum }_{j=1}^{M}{p}_{j}(1-{p}_{j})}, h\ne i$$

where ${\eta }_{j}=1-4{p}_{j}\left(1-{p}_{j}\right)$ and ${S}_{ij}={Y}_{ij}/{d}_{ij}$. An adjustment for the self-relatedness estimates is required and is computed by

15 $${G}_{ii}={\psi }_{i}\frac{{\sum }_{j=1}^{M}\left[{\left({S}_{ij}-{p}_{j}\right)}^{2}-{A}_{ij}\right]/{(\gamma }_{j}{\delta }_{ij}) }{{\sum }_{j=1}^{M}{p}_{j}(1-{p}_{j})}, {d}_{ij}\in \{2,3,\hdots\}$$

where

16 $${A}_{ij}={p}_{j}\left(1-{p}_{j}\right)\left(1-{\gamma }_{j}{\delta }_{ij}\right)+{\varepsilon }_{j}\left({\eta }_{j}-\left(1-{\varepsilon }_{j}\right){\delta }_{ij}\right).$$

See Supplementary File 1 for full derivations of Eqs. (14) and (15). Note that the adjustment for self-relatedness is only computed when the read depth is 2 or more, since read depths of 0 and 1 do not contain any information about inbreeding (Dodds et al. 2015).

#### Missing data

The relatedness estimators derived in the previous section assume that there are no missing genotypes or that there is at least one read associated with each genotype for HTS data. In practice, missing genotypes often occur in genotypic data while HTS data typically contains a large proportion of missing genotype calls due to there being no corresponding reads, especially at low read depths. Often missing genotypes are replaced with the mean population value, but this introduces bias and leads to underestimation of relatedness (Goudet et al. 2018; Horton and Kleinman 2007; Jarquin et al. 2014). More sophisticated imputation methods are available that result in less bias but require additional information such as marker ordering which is often not available (Marchini and Howie 2010). An alternative approach is to assume that data are missing at random and estimate relatedness using only SNPs with non-missing genotype information for each pair of individuals or within an individual (Dodds et al. 2015; Goudet et al. 2018; VanRaden 2008). Dodds et al. (2015) developed such an approach for diploids that utilizes matrix computation for efficient calculation of GRM estimators with missing data. We extend their approach to the polyploid GRM estimators we have derived here (see Supplementary File 1 for details).

### GUSrelate

An implementation of the new GRM estimator we have derived in this article is available in the R package GUSrelate (genotyping uncertainty with sequencing data for relatedness) which can be downloaded at https://github.com/tpbilton/GUSrelate. GUSrelate also contains additional functionality for examining the GRM estimates and to output the GRM to a file. An introduction on how to use GUSrelate along with its features and workflow is available from https://github.com/tpbilton/GUSrelate/blob/master/README.md.

### Methods comparison

Using simulated data and a real HTS dataset, we compared the performance of GUSrelate to two alternative approaches for constructing GRMs based on the VanRaden approach. The approaches we considered were the ratio method implemented in the *R* package AGHmatrix v2.1.3 (Amadeu et al. 2016), and the method proposed by Cericola et al. (2018) developed for pooled HTS data. The function Gmatrix in the AGHmatrix package with ratio = TRUE was used to construct the GRMs, whereas the Cericola et al. method was implemented using custom *R* code.

### Simulation study

**Fig. 1:**
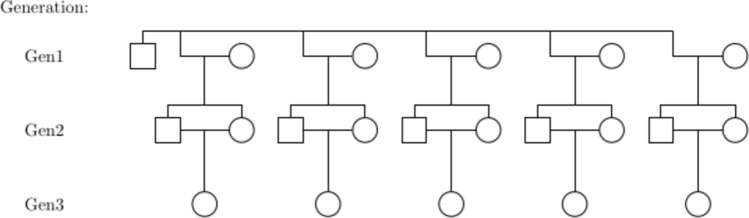
Structure of each independent family from which the pedigree in the simulation study was constructed. Squares represent paternal parent, circles represent maternal parent, lines denote relationships. The pedigree used in the simulation study was made up of 20 independent families of this structure

Simulation of data for comparing the different GRM methods proceeded as follows. A population was set up that consisted of 20 unrelated families with the structure (see Fig. 1) of one male individual being mated with 5 unrelated female individuals (Gen1) to produce two offspring each (Gen2) that mate to produce one inbred individual (Gen3). The number of individuals in the pedigree is therefore 420. This pedigree structure was used to give a large number of specific relationships (e.g. half-sibs, full-sibs) present in the relationship matrix but also to allow some variation in average relationship values between individuals. The different relationships present in the pedigree that will be evaluated in the simulation are given in Table 1.

**Table 1:** Specific relationships in pedigree used in the simulation

Relationship	Abbreviation	Number of pairs in pedigree	Expected relatedness^a^
Self-relatedness (Gen1)	SR (G1)	120	1
Self-relatedness (Gen2)	SR (G2)	200	1
Self-relatedness (Gen3)	SR (G3)	100	1.25
Parent-offspring (Gen2–Gen3)	PO (G2–G3)	200	0.75
Parent-offspring (Gen1–Gen2)	PO (G1–G2)	400	0.5
Full siblings (Gen2)	FS (G2)	100	0.5
Grandparent-offspring (Gen1–Gen3)	GO (G1–G3)	200	0. 5
Half siblings (Gen2)	HS (G2)	800	0.25
Half cousins (Gen3)	HC (G3)	200	0.25
Pibling^b^-half nibling^c^ (Gen2–Gen3)	PHN (G2–G3)	800	0.25
Unrelated	UR	85290	0

^a^Expected relatedness in diploids or in polyploids based on pedigree and assuming no double reduction

^b^Pibling is a gender-neutral word that refers to uncle or aunty

^c^Nibling is a gender-neutral word that refers to niece or nephew

The founders of this population (parents of Gen1) were assumed to be unrelated (all alleles are non-IBD) and binomial sampling (i.e. ${X}_{ij}\sim {\text{Bin}}({\psi }_{i},{p}_{j})$) was used to simulate genotypes of these founders for each SNP (assuming independence between SNPs for the founders), where founder allele frequencies, ${p}_{j}$, for each SNP were sampled from a uniform distribution on [0,1]. SNP positions were determined by spacing SNPs at equal centimorgan distances across 12 chromosomes, with the number of SNPs per chromosome being in proportion to the total centimorgan length of the 12 chromosomes in potato as estimated by Bourke et al. (2015). Potato was used as a benchmark for simulating data to match the real dataset used later in this study. Genotype data were then simulated using PedigreeSim v2.2 (Voorrips and Maliepaard 2012) with default settings and the Kosambi mapping function, for various ploidy levels. The centromere for each chromosome was based on the estimates from Bourke et al. (2015) and were averaged across the two parental lines.

Sequencing data were simulated from the genotype data in two steps. (1) generate a read depth for each genotype call by simulating realizations from a negative binomial distribution with mean ${\mu }_{{d}_{j}}$ and dispersion parameter equal to the ploidy ${\psi }_{i}$:

17 $$P\left({d}_{ij}=d\right)= \frac{\Gamma \left(d+{\psi }_{i}\right)}{d!\Gamma \left({\psi }_{i}\right)}{\left(\frac{{\psi }_{i}}{{\mu }_{{d}_{j}}+{\psi }_{i}}\right)}^{{\psi }_{i}}{\left(\frac{{\mu }_{{d}_{j}}}{{\mu }_{{d}_{j}}+{\psi }_{i}}\right)}^{d},$$

where ${\mu }_{{d}_{j}}$ is the mean read depth for SNP $j$ and $\Gamma \left(\cdot \right)$ denotes the gamma function. (2) randomly sample the alleles in each true genotype with replacement using a sample size of ${d}_{ij}$ and allowing for a miscalled allele with probability ${\varepsilon }_{j}$ (i.e. sample according to Eq. (9)). The negative binomial distribution was chosen as it has been used in previous HTS simulation studies (e.g. Bilton et al. (2018a), Bilton et al. (2018b)).

Two simulations were performed. For the first simulation, 1000 datasets were simulated for a given set of parameters (ploidy, mean read depth and sequencing error rate) using the pedigree described previously (Fig. 1) and fixing the number of SNPs to 10,000. Different combinations of the parameters were used where the ploidy was the same for all individuals but either diploid (${\psi }_{i}=2$), tetraploid (${\psi }_{i}=4$), hexaploid (${\psi }_{i}=6$) or octaploid (${\psi }_{i}=8$), the average read depth was either low (${\mu }_{{d}_{j}}$ = 5), moderate (${\mu }_{{d}_{j}}$ = 25) or high (${\mu }_{{d}_{j}}$ = 50), and the sequencing error rate was either absent (${\varepsilon }_{j}=0$), small (${\varepsilon }_{j}=0.001$), or large (${\varepsilon }_{j}=0.01$). GRMs were constructed using GUSrelate with no sequencing error (GUS), GUSrelate using the true sequencing error rate (GUS_err), AGHmatrix (AGH) and the method by Cericola et al. (2018) (Cer). The performance of each method was assessed by computing the root mean squared error (RMSE) of the self-relatedness estimates ($((1/N){\sum }_{i=1}^{N}({G}_{ii}-{\widehat{G}}_{ii}{)}^{2}{)}^{(1/2)}$) and the relatedness estimates ($((1/N){\sum }_{h=1}^{N}{\sum }_{i=1,i\ne h}^{N}({G}_{hi}-{\widehat{G}}_{hi}{)}^{2}{)}^{(1/2)}$), where the “true” self-relatedness value (${G}_{ii}$) and relatedness value (${G}_{hi}$) were determined by computing the true simulated IBD probabilities using the simulated IBD states of the SNPs, which were extracted from the output of PedigreeSim. These IBD probabilities represent the “true relationships” and will vary between individuals with the same pedigree relationship as there will be variation due to double reduction and Mendelian sampling. This means that the true relationships account for Mendelian sampling. The bias and variance of the GRM estimates were also examined to investigate performance of the estimators.

The second simulation was performed to investigate the optimal sequencing depth when the sequencing effort (defined as the number of individuals times the number of SNPs times the mean read depth) is fixed. In this simulation set, 500 datasets were simulated for average read depths ranging from 1 to 40, where the sequencing error rate was fixed at 0.1% and the number of SNPs set to maintain a sequencing effort of 10 million reads (i.e. $N\times M\times {\mu }_{{d}_{i}}=\mathrm{10,000,000}$) and 40 million reads (see Supplementary Table S1 for parameter values used in this simulation set). GRMs were constructed in GUSrelate using the true sequencing error rate of 0.1%, and the RMSE calculated as in the first simulation.

### Potato dataset

Potato datasets were generated using 351 tetraploid clones (*Solanum tuberosum* L.) as described in Sharma et al. (2018). Two types of genotyping datasets (SNP array and GBS) were obtained, as described in the subsequent sections. Both datasets were used to compare the various GRM estimators of relatedness.

#### SNP array

SNP array data for 351 tetraploid potato clones were generated (Sharma et al. 2018). The cultivar ‘Adirondack’ was used as a control across all 16 Infinium 8k Potato SNP array (Felcher et al. 2012; Hamilton et al. 2011) genotyping batches of 24 genotypes each. SNP allelic dosages (genotypes) were called using the *R* package fitPoly v3.0.0 (Voorrips et al. 2011). SNPs reported as mapping to multiple locations on the potato genome by Hirsch et al. (2013) and SNPs with inconsistent genotype calls or at least 30% missing genotypes across the 16 control samples were excluded (2537 in total). Further SNP filtering was performed with SNPs discarded if 20% or more of the genotype calls were missing across all samples, but no SNPs were filtered based on minor allele frequency (MAF), as the minimum MAF was 0.03 across all SNPs. In total, 5514 SNPs remained after filtering. This SNP dataset is called ‘Geno’ in the GRM estimator comparisons.

#### Genotyping-by-sequencing

Sequence data from sixteen 24-plex ‘*Pst*I-*Mse*I’ GBS libraries (384 samples in total), constructed using the procedure adapted from Poland et al. (2012), were obtained (Sharma et al. 2023). The data comprised 150 bp single-end sequence reads derived using Illumina HiSeq 2500 runs, with one 24-plex GBS library per single flow cell lane. GBS data were deconvoluted into single sample reads and quality trimmed using in-house Java and shell scripts. The read data for 351 tetraploid samples (same as for the SNP array data described above) was extracted and mapped onto the potato reference genome version 4.03 (the doubled monoploid potato *S*. tuberosum Group Phureja DM 1-3 516 R44) (Sharma et al. 2013; The Potato Genome Sequencing 2011) using Bowtie2 (Langmead and Salzberg 2012) followed by variant discovery using freeBayes v0.9.14-14-gb00b735 (Garrison and Marth 2012) keeping the default settings except for the following:

*“–ploidy 4 –no-population-priors –min-alternate-count 4 –min-alternate-fraction 0.15 –min-alternate-total 4 –genotype-qualities –report-genotype-likelihood-max –use-mapping-quality –min-mapping-quality 10 –max-complex-gap 130 –haplotype-length 130 –min-coverage 16”*. The analysis resulted in 270,358 raw SNPs, with filtering performed as follows. SNPs were discarded if the MAF < 0.01, 50% or more genotypes were missing (i.e. with a read depth of zero), or the average SNP read depth was > 500 (150,190 in total). A Hardy–Weinberg equilibrium (HWE) test was performed using an extension of the HWE test for HTS data described by Li (2011) to autopolyploids (see supplementary materials for details), with SNPs discarded if the *p*-value from the HWE test was < 0.1 (76,070 in total). This filter removes SNPs that had increased observed homozygosity or heterozygosity relative to what was expected under HWE. The remaining SNPs were divided into two SNP subsets: a low-depth set (14,843 in total) and a high-depth set (29,255 in total) depending on whether the average SNP read depth was below or above 50. GRMs were constructed on these two SNP subsets to examine the performance of the various GRM estimators in both a low and high read depth setting.

## Results

### Simulation study

**Fig. 2:**
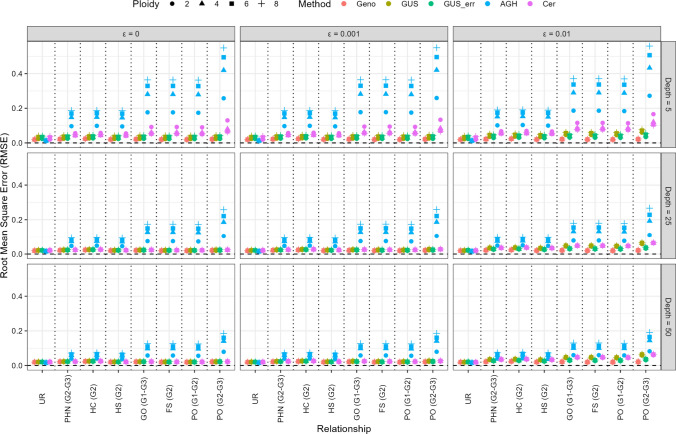
Root mean square error (RMSE) of relatedness (off-diagonal) estimates for different GRMs. Each point represents the average RMSE across 500 datasets simulated using the specific combination of sequencing error, mean read depth and ploidy level, and for pairwise relationship specified in Table 1. Rows represent datasets simulated with average read depth of 5 (top row), 25 (middle row) and 50 (bottom row), columns represent datasets simulated with no sequencing error (first column) and a mean sequencing error of 0.1% (middle column) or 1% (right column). Points are coloured based on method used to construct the GRM, where the methods used were GUSrelate with no sequencing error (GUS) and using the true sequencing error rate (GUS_err), AGHmatrix (AGH) and Cericola et al. (2018) (Cer), which are compared to estimates from the GRM constructed using the true genotypes (Geno). The *x*-axis denotes whether the relationship between individuals was unrelated (UR), pibling-half nibling for Gen2–Gen3 (PHN (G2–G3)), half cousins for Gen3 (HC (G3)), half siblings for Gen2 (HS (G2)), grandparent-offspring for Gen1–Gen3 (GO (G1–G3)), full sibling in Gen 2 (FS (G2)), Gen1–Gen2 (PO (G1–G2)), or parent–offspring for Gen2–Gen3 (PO (G2–G3)), and different symbols are used to denote whether the ploidy was diploid (circle), tetraploid (triangle), hexaploid (square) or octoploid (cross) species

The RMSE for the relatedness estimates (off-diagonal elements) from the different methods is given in Fig. 2 for the different relationships specified in Table 1. Note that the relatedness estimates from the SNP array data (Geno) shown in Fig. 2 are the same for each combination of read depth and sequencing error. For the AGHmatrix package, the RMSE was considerably larger than for GUSrelate in all scenarios, where the RMSE was larger at lower average read depths and when the expected relationship value was larger (e.g. largest RMSE for PO (G2–G3) which has the largest expected relatedness value). Similarly, the RMSE for the Cericola et al. method was considerably larger compared to GUSrelate at a low mean read depth (${\mu }_{{d}_{ij}}=5$) or when the sequencing error rate was large (${\varepsilon }_{j}=0.01$). Nevertheless, this increase in the RMSE when benchmarked against GUSrelate was smaller than the increase for AGHmatrix. At moderate-to-high average read depths and low to no sequencing error, the RMSE was relatively low for the Cericola et al. method and was similar to the RMSE for GUSrelate with or without sequencing errors accounted for in the estimation. GUSrelate was the only HTS method that had consistently low RMSE at low depths, and GUSrelate accounting for sequencing errors (GUS_err) was the only method that showed minimal increase in the RMSE at the high sequencing error rate of 1%. When compared to RMSE from using the true genotypes (Geno), the RMSE for GUSrelate was similar at high read depth but had a small increase as the average read depth decreased, as there is more variability in the relatedness estimates at lower depths. The RMSE also increased with ploidy level across all methods for sequencing data, which is due to there being less information about the true genotype in HTS data for a fixed number of reads. For all the GRM methods used on HTS data at low to moderate depths, the relatedness estimates were more variable compared to the genotypic GRM (Geno) (Supplementary Figure S2). On the other hand, the relatedness estimates from AGHmatrix and Cericola et al. both had a larger negative bias compared to GUSrelate (Supplementary Figure S1). This indicates that for relatedness estimates, the difference in RMSE between the methods for HTS data was driven by differences in the bias of the relatedness estimates.

**Fig. 3:**
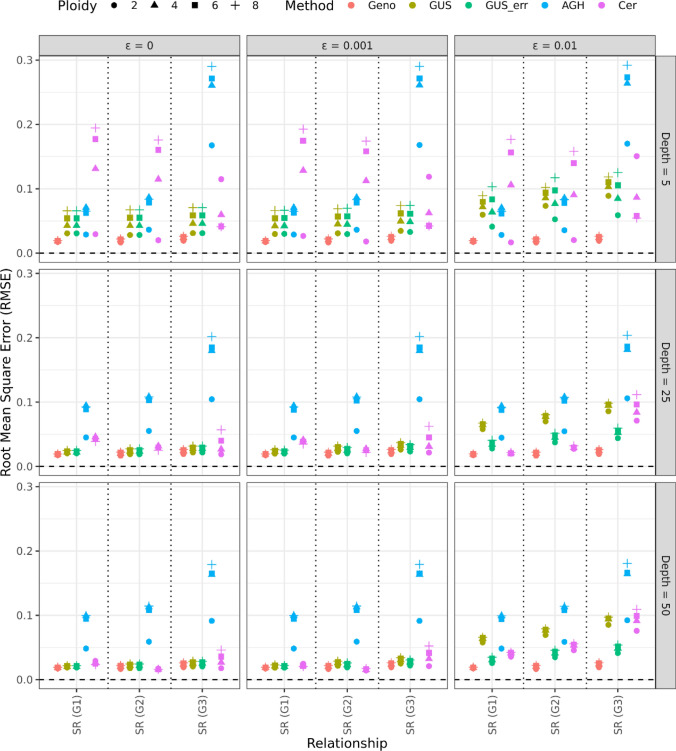
Root mean square error (RMSE) of self-relatedness (diagonal) estimates for different GRMs. Each point represents the average RMSE across 500 datasets simulated using the specific combination of sequencing error, mean read depth and ploidy level, and for different generations. Rows represent datasets simulated with average read depth of 5 (top row), 25 (middle row) and 50 (bottom row), columns represent datasets simulated with no sequencing error (first column), and a mean sequencing error of 0.1% (middle column) or 1% (right column). Points are coloured based on method used to construct the GRM, where the methods used were GUSrelate with no sequencing error (GUS) and using the true sequencing error rate (GUS_err), AGHmatrix (AGH) and Cericola et al. (2018) (Cer), which are compared to estimates from the GRM constructed using the true genotypes (Geno). The x-axis denotes whether the relationship between individuals was for individuals in generation 1 (SR (G1)), generation 2 (SR (G2)) or generation 3 (SR (G3)), and different symbols are used to denote whether the ploidy was diploid (circle), tetraploid (triangle), hexaploid (square) or octoploid (cross) species

The RMSE for the self-relatedness estimates (diagonal GRM elements) for the three different generations in the pedigree from the different methods is given in Fig. 3. The results were similar to the those for the off-diagonal GRM estimates, with AGHmatrix having higher RMSE in self-relatedness estimates compared to the other methods.

**Fig. 4:**
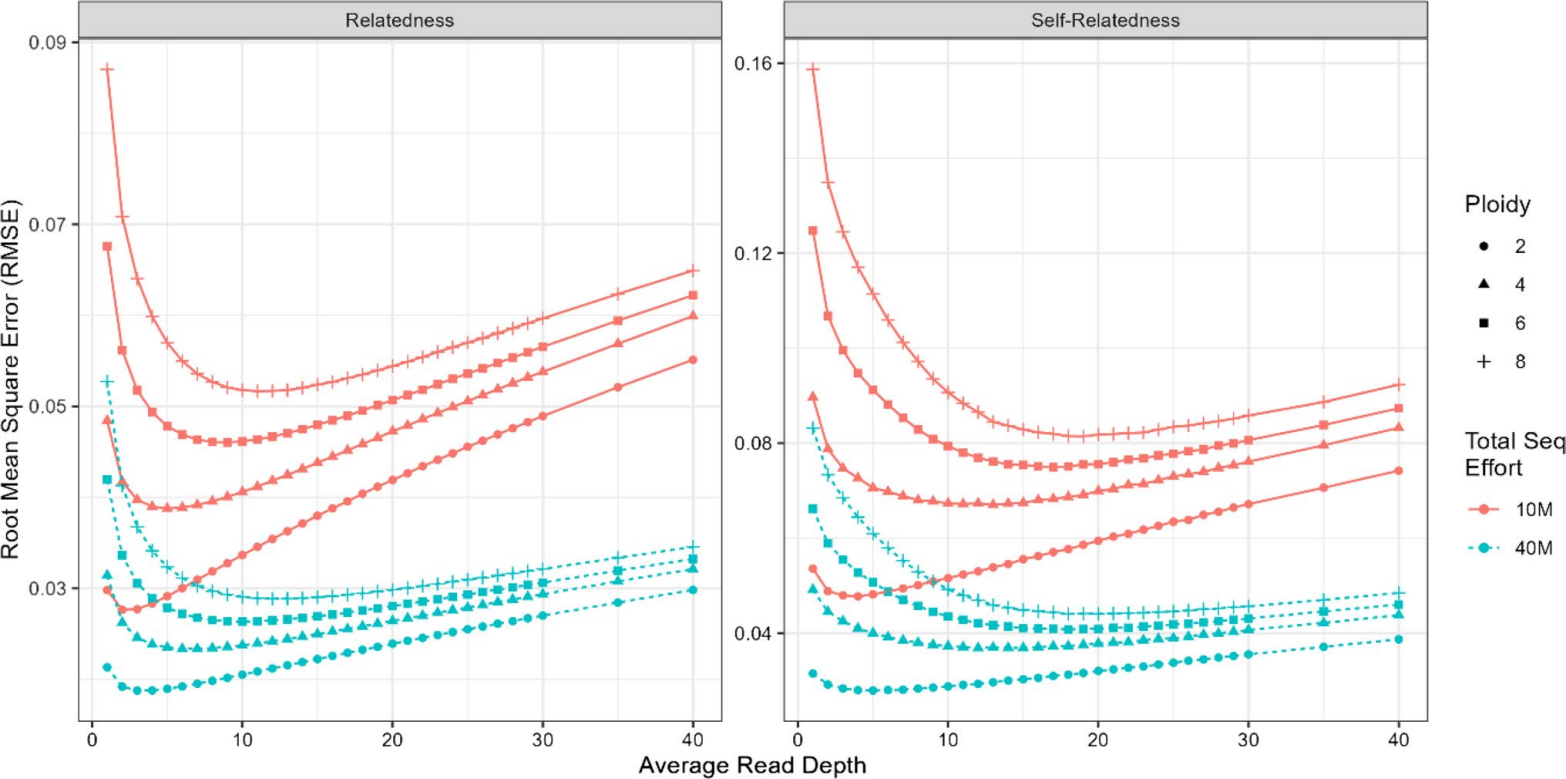
Root mean squared error (RMSE) of the GRM relatedness (left panel) and self-relatedness (right panel) estimates for the simulation using a fixed sequencing effort. The parameters used to generate the datasets were varied, where the ploidy level was 2 (diploid; circle), 4 (tetraploid; triangle), 6 (hexaploid; square) and 8 (octoploid; plus), the mean depth ranged from 1 to 40, and the number of SNPs was set to maintain a sequencing effort of 10 million reads (solid red line) and 40 million reads (dashed blue line). See Table S1 for the number of SNPs used for each set of parameters. The sequencing error rate (0.1%) and the number of individuals (420) were fixed in the simulation. The GRM estimates were computed using GUSrelate assuming the true sequencing error rate of 0.1%. Each point denotes the RMSE average across 500 datasets

Figure 4 gives the average RMSE of the relatedness and self-relatedness estimates from the GRM computed using GUSrelate in the second simulation versus the mean read depth. From this figure, we see that the optimal depth (lowest RMSE) for diploids was around 2–3 for relatedness estimates and 4 for self-relatedness estimates when the total sequencing effort was 10M reads. As the ploidy level increased, the optimal depth for GRM relatedness estimates also increased, with an optimal depth around 5 (tetraploids), 9 (hexaploids) and 11–12 (octoploids) for relatedness estimates, and an optimal depth around 13 (tetraploids), 17 (hexaploids) and 18–19 (octoploids) for self-relatedness estimates when the total sequencing effort was 10M reads. These results are likely explained by the fact that there are more alleles in each genotype for higher ploidy levels, so more reads are required to obtain the same level of precision in inferring the genotypes. When the total sequencing effort was increased to 40M reads, the optimal read depth increased by 1–2 across all combinations of ploidy and relationship (i.e. relatedness/self-relatedness). The RMSE also increased more rapidly at very low read depths (1–5) compared to read depths greater than 25 as the ploidy level increased. This suggests that having sufficient depths is important in estimating relatedness with HTS, especially for species with higher ploidy levels.

### Potato dataset

**Fig. 5:**
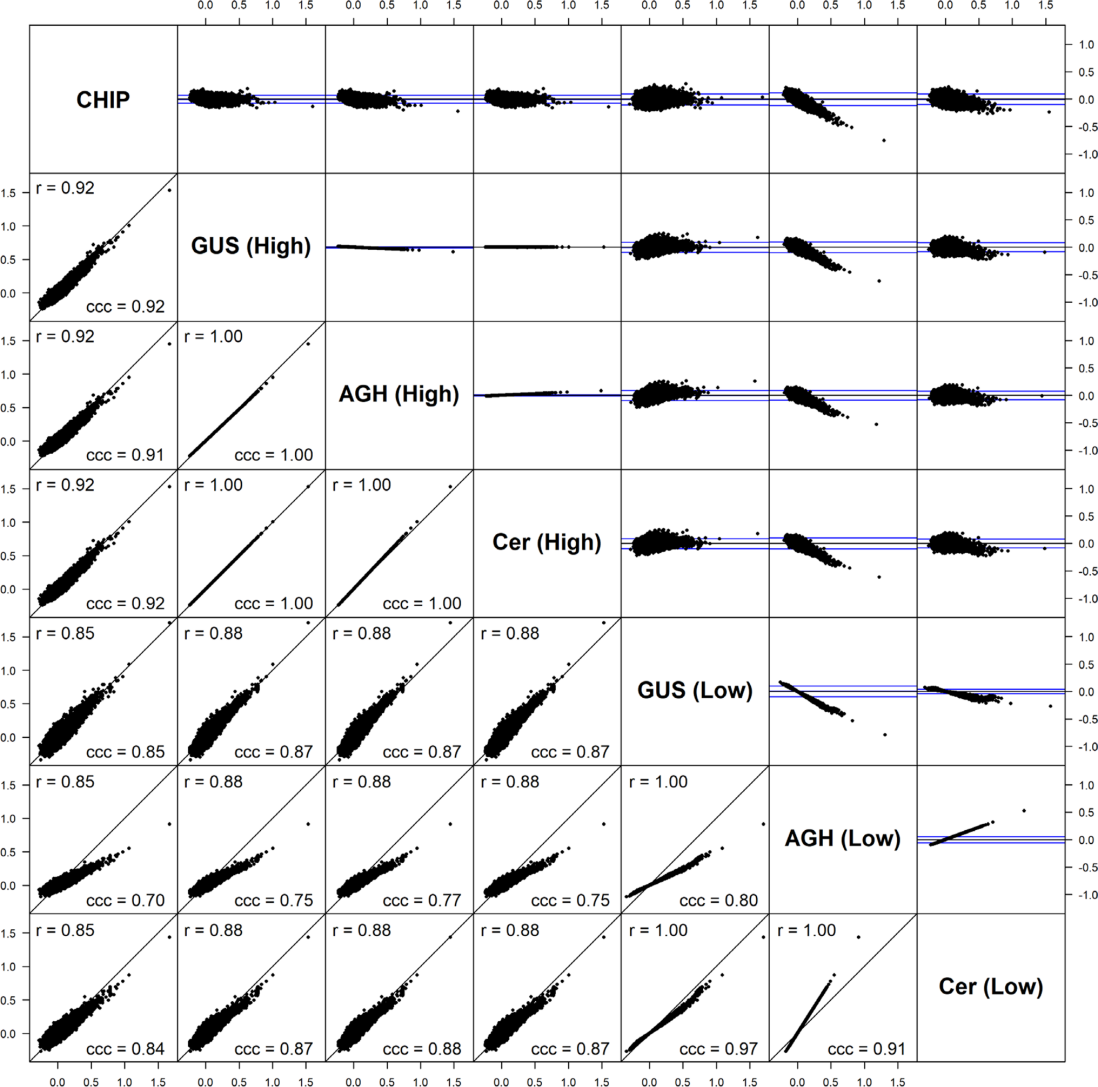
Matrix plot comparing the relatedness (off-diagonal) estimates between different GRMs. Lower diagonals show scatter plots of relatedness estimates between two GRMs along with Spearman's correlation coefficient (r) and Lin’s concordance correlation coefficient (ccc) for each pair of GRMs, while the upper diagonals show the corresponding Bland–Altman plots. The combination of dataset and method used to construct the GRM is specified on the diagonal and correspond to the codes given in Table 2

**Fig. 6:**
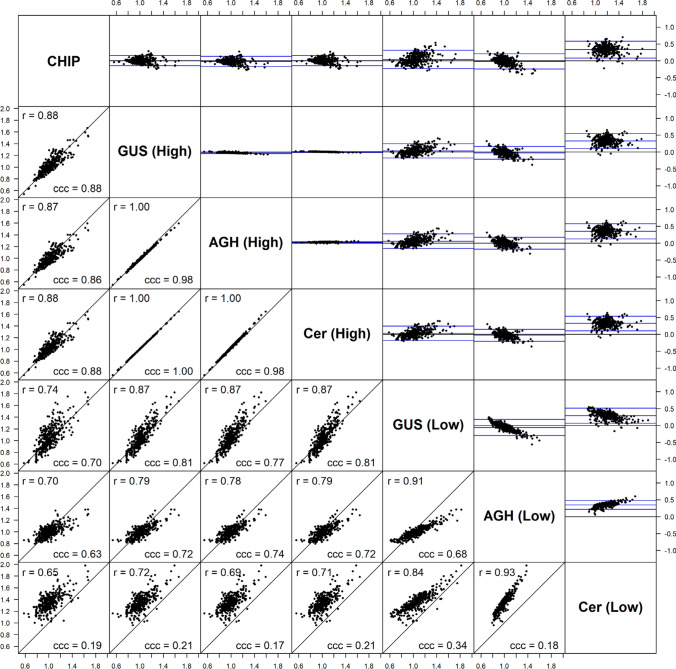
Matrix plot comparing the self-relatedness (diagonal) estimates between different GRMs. Lower diagonals show scatter plots of self-relatedness estimates between two GRMs along with Spearman's correlation coefficient (r) and Lin’s concordance correlation coefficient (ccc) for each pair of GRMs and the upper diagonals show the corresponding Bland–Altman plot. The combination of dataset and method used to construct the GRM is specified on the diagonal, and correspond to the codes given in Table 2

GRMs for the potato population were constructed from the SNP array data using the estimator in Eq. (6) and from the GBS data using combinations of the low- and high-depth SNP sets with the GUSrelate, AGHmatrix and Cericola et al. (2018) methods (see Table 2). A matrix plot showing all pairwise comparisons of the GRM estimates is given in Fig. 5 for the relatedness values (off-diagonal elements) and Fig. 6 for the self-relatedness estimates (diagonal elements). The lower diagonals of the matrix plots are scatter plots of the GRM estimates between two methods, and the corresponding Bland–Altman plots (Bland and Altman 1999) are given in the upper diagonals. The Bland–Altman plots show the difference between GRM estimates (*y*-axis) relative to the average of two estimates (x-axis) and has two blue horizontal lines representing two standard deviations above and below the line of no difference. The plot is useful for showing bias and how closely estimates agree between two methods.

**Table 2:** Combinations of datasets and methods used to construct the GRM for the potato population

Key	Genotyping Platform	Method	Average read depth
CHIP	SNP array	Equation (6)	N/A
GUS (High)	GBS	GUSrelate	> 50
AGH (High)	GBS	AGHmatrix	> 50
Cer (High)	GBS	Cericola et al. (2018)	> 50
GUS (Low)	GBS	GUSrelate	< 50
AGH (Low)	GBS	AGHmatrix	< 50
Cer (Low)	GBS	Cericola et al. (2018)	< 50

The relatedness and self-relatedness estimates for the high-depth SNP set across all three methods, GUSrelate (GUS (High)), AGHmatrix (AGH (High)) and Cericola (Cer (High)), were highly correlated, and were also strongly correlated with the GRM estimates from the SNP array data (CHIP). This observation is expected, as at depths greater than 50, the effect of low read depth on relatedness estimates is minimal, meaning that the different methods provide very similar GRMs. The GRM constructed from the low-depth SNP set using AGHmatrix (AGH (Low)) has self-relatedness estimates biased downwards and relatedness estimates biased towards zero compared to the SNP array GRM. This is due to the AGHmatrix package using the sample variance estimated from the data instead of ${\sum }_{j=1}^{M}{\widehat{p}}_{j}(1-{\widehat{p}}_{j})$ in the denominator of their estimator by default, which results in an inflation of the denominator value since the sample variance of the allele frequencies increases as the read depth decreases. On the other hand, the GRM estimates from GUSrelate at low depth (GUS (Low)) were still highly correlated with the estimates from the SNP array data (CHIP), although this correlation was slightly lower than in the high read depth setting. The Cericola method performed better than AGHmatrix at low read depth but still had self-relatedness estimates that were considerably larger than, and compared to GUSrelate less correlated with, the estimates from the SNP array data.

## Discussion

The introduction of HTS methods has dramatically improved the feasibility of conducting genetic studies in autopolyploid species, as it has reduced the cost of sequencing and removed the need for a discovery phase such as is required for SNP arrays. Consequently, the number of studies using HTS methods on autopolyploid species has increased rapidly in recent years. For many of these studies, a GRM is constructed for use in various applications, such as population analysis, genomic selection, genome-wide association studies and pedigree analysis. To date, most autopolyploid studies in the literature have used some form of the VanRaden (method 1) GRM estimator that has been extended to autopolyploid species. In this article, we have provided a mathematical derivation of the mixed-ploidy autopolyploid version of the VanRaden (method 1) GRM estimator that confirms the formulations found in the literature. Our derivation also provides some insight into properties of the estimator for the polyploid setting. In particular, it shows that by construction, the GRM estimator accounts for all forms of inbreeding but is unable to distinguish inbreeding due to double reduction from inbreeding resulting from mating related individuals. This highlights another advantage of genomic estimates of relatedness over pedigree-based relatedness estimates. Relatedness estimates from the pedigree-based numerator relationship matrix (Hamilton and Kerr 2018; Kerr et al. 2012; Slater et al. 2014) generally require a fixed genome-wide double reduction rate value to be prespecified in the calculations. In practice, the true double reduction rate may be unknown, especially for novel or under-studied species, while the double reduction rate is known to vary across a chromosome with the probability of a double reduction event being zero at the centromere and increasing with increasing distance from the centromere (Voorrips and Maliepaard 2012). The GRM estimator, on the other hand, bypasses these limitations as it automatically captures variation of inbreeding, and therefore double reduction, across the genome since this is contained in the genotypic information.

Our main contribution in this manuscript is the development of a GRM relatedness estimator that accounts for errors due to low sequencing read depth and miscalled bases (i.e. sequencing errors), which we have implemented in the R package GUSrelate. This estimator is based on the VanRaden polyploid estimator and can be considered as an extension of the diploid GRM estimator for HTS data developed by Dodds et al. (2015) to polyploids and includes accounting for sequencing errors. Our results, both mathematically and from the data analysis, indicate that self-relatedness estimates from existing methods are increasingly biased upwards as average read depth decreases, whereas relatedness (off-diagonal GRM elements) values are relatively unaffected by low read depths. These observations are consistent with GRM estimation in diploids using low-depth HTS as observed by Dodds et al. (2015) and Ashraf et al. (2016). On the other hand, our results indicate that sequencing errors impact both the self-relatedness and relatedness estimates of the GRM, resulting in a downward bias that increases with increasing sequencing error rates when not accounted for in the GRM estimation. A likely explanation of this observation is that sequencing errors will result in, on average, more homozygous genotypes being wrongly called as heterozygous than vice versa which will decrease (self)-relatedness values when not taken into account in the computation of the GRM.

Using simulations and a real dataset, we compared our new method, GUSrelate, to two alternative methods found in the literature, which were the default method implemented in the AGHmatrix package and the method by Cericola et al. (2018). We found that AGHmatrix resulted in considerable bias in the relatedness estimates with low-depth HTS data and would recommend sequencing and filtering to a reasonably high depth if employing this method. This bias for AGHmatrix is due to it using the sample variance to estimate the denominator of the VanRaden estimator which is inflated in the presence of low read depths resulting in relatedness estimates being biased towards zero. Furthermore, AGHmatrix does not account for the inflation of self-relatedness estimates due to low read depths. The Cericola et al. (2018) method, on the other hand, was designed for pooled samples and provides an adjustment to the diagonals of the VanRaden GRM. Although this method performed better than AGHmatrix in our analysis, we found the self-relatedness estimates were still biased, although the average self-relatedness was approximately similar to that from the GRM from genotypic data. In contrast, GUSrelate performed well, with lower RMSE in the simulations compared to the other methods, and had estimates that were more similar to those obtained using genotypes from a SNP array in a potato population compared to AGHmatrix and the Cericola et al. method.

A limitation of our GRM estimator is that the ancestral frequencies are assumed known when in fact they must be estimated. This limitation is not unique to our estimator and is a common problem for most relatedness estimators. Frequently, the study population is used to estimate the allele frequencies although this is known to introduce bias in the relatedness estimates (Wang 2014). This practice is widely accepted and is a reasonable approximation provided that the population is relatively unrelated on average (Milligan 2003). In addition, the SNP specific sequencing error rates, ${\varepsilon }_{j}$, in our estimator are assumed to be constant across individuals and known prior to constructing the GRM. In practice, ${\varepsilon }_{j}$ would need to be estimated from the data, which is not straightforward, and biased estimates have been shown to occur in other types of genetic analyses involving HTS data when the read depth is low (Bilton et al. 2018a, 2018b). One possible approach to obtain sequencing error values would be to extract the Phred-scaled quality scores associated with each genotype call from the VCF file (e.g. using the QR and QA fields which give the sum of the Phred-scaled quality scores for the reference and alternate alleles) and back transform to obtain error probabilities. Alternatively, error probabilities could be estimated from suitable genotyping software, such as updog (Gerard et al. 2018), although this would add extensive computational time to obtaining the relatedness estimates. However, our simulation results suggest that the bias resulting from sequencing errors is relatively low in estimating relatedness, implying that the impact on results is small. One pragmatic solution, therefore, is for the user to pre-specify the sequencing error based on previous experience with HTS data. Previous HTS studies have found that the overall sequencing error rate to be between 0.1% and 0.3% (Bilton et al. 2018b; Clark et al. 2019; Pfeiffer et al. 2018).

A number of assumptions underpin the GRM estimators developed in this article. The first is that an offspring has equal chance of inheriting any set of the alleles found in each of their parents. This means that the estimators are not applicable to segmental polyploids where some degree of preferential pairing occurs. Additionally, this assumption also implies the absence of genetic forces acting on the population, such as selection. Another assumption is that the alleles in the genotype are sampled randomly according to the binomial model in Eq. (9). In reality, overdispersion in the allele counts has been observed in HTS data, particularly in polyploids (Clark et al. 2019; Dodds et al. 2019; Gerard et al. 2018). Our estimator could be extended to accommodate alternative sampling models following similar derivations, provided that an analytical expression for the mean and variance of the sampling distribution can be derived. One alternative model used in the literature is the beta-binomial model (Clark et al. 2019; Dodds et al. 2019; Gerard et al. 2018). This model has the same mean but larger variance than the binomial model, which means that under this model relatedness estimates will remain unchanged but self-relatedness values will increase due to the larger variance. The caveat is that the beta-binomial model has an extra (dispersion) parameter that, like the sequencing error rate, would require estimation prior to constructing the GRM, meaning that an investigation into the best approach for this would be required. The binomial model also implies that each allele has equal chance of being sampled, whereas preferential sampling of one allele over the other (i.e. allelic bias), due to uneven amplification or issues with read alignment, has been observed in practice (Furuta et al. 2023; Gerard et al. 2018). The presence of allelic bias will result in inflated self-relatedness estimates as genotypes will be more homozygous than expected under the binomial model. On the other hand, we expect that allelic bias is unlikely to affect relatedness estimates provided the bias is consistent across the data. This is because any bias in relatedness estimates tends to cancel out as seen with our estimators where the read depths are not required in the relatedness estimator for low-depth sequencing data. In practice, bias in self-relatedness estimates due to allelic bias can be mitigated by applying appropriate SNP filtering to remove SNPs with a high degree of allelic bias. A further assumption is the ploidy level of each individual in the population is known. In scenarios where this is not the case, methods are available for determining ploidy level in HTS data (e.g. Gompert and Mock (2017)) prior to GRM construction. Lastly, the GRM implemented in GUSrelate assumes that missing data occurs at random. This is a common assumption of many GRM estimators (Dodds et al. 2015; Goudet et al. 2018) and simplifies the process of constructing the GRM. What effect violation of the missing at random assumption has on the GRM estimator is unknown, although it seems a reasonable assumption in practice.

One issue with analysing HTS data is that filtering is required to remove problematic SNPs, as these can negatively impact analyses. In diploid species, suitable methods for filtering out problematic SNPs have been developed and are well tested but do not readily transfer to autopolyploid species. In this study, we used a HWE filter in an attempt to remove problematic SNPs. We found that this filter improved the relatedness estimates, particularly for the low-depth SNPs where the self-relatedness estimates were nearly double and the relatedness values were biased by a factor of a half when no HWE filter was used compared to the results obtained using the HWE filter (see Figs. S5 and S6 in Supplementary File 2).

However, the HWE filter is not ideal, as it is likely to remove some real SNPs and bias the GRM estimates. One case of this is for SNPs where a large number of double reduction events are present, which results in a distortion of the genotype frequency distribution under HWE. Removing these SNPs could result in downwardly biased self-relatedness and relatedness estimates, as SNPs with more double reduction events have higher levels of inbreeding compared with SNPs with no double reduction events. More appropriate methods for filtering out problematic SNPs in polyploids are needed. A recent Bayesian method for testing random mating in autopolyploids developed by Gerard (2023) is a possible alternative that might be a more appropriate method for filtering SNPs as it accounts for double reduction in its test. The effect these filtering methods have on relatedness estimation should be examined.

There is scope to extend our estimators to other applications. One application is estimating relatedness for pooled samples, where the ploidy level is equivalent to the total number of gametes in the pool (i.e. the sum of ploidy level of all species in the pool), and has been explored by Cericola et al. (2018). However, this requires that both the number of individuals in the pool and the ploidy level of individuals are known. Another application is to use our GRM estimator for parentage assignment using a similar approach to that employed by Dodds et al. (2019). This could be useful for completing partial pedigrees or for checking the quality of existing pedigrees for errors. However, methods for parentage assignment in polyploids are limited compared to diploids, and further research into this topic would be required. A third potential application is to use the GRM estimator to determine approximately the location of the centromere on a chromosome. This would be possible as the GRM estimator (both the genotypic and HTS version) captures the variation of inbreeding across the genome as discussed earlier. To achieve this, a per-chromosome sliding window approach would need to be employed whereby the GRM is re-estimated using only SNPs within the window and the inbreeding estimates plotted against the window position on the chromosome. This would require a quality reference genome for the species of interest to correctly determine all SNP positions, and a large number of SNPs distributed across the entire chromosome. An investigation into how large a population size is sufficient to detect the centromere would be needed.

An important consideration for researchers planning to conduct HTS studies on polyploid species is the average read depth to aim for when sequencing. Sequencing depth in autopolyploid populations is a controversial topic and highly stringent deep sequencing often recommended in the literature can be cost prohibitive and, depending on the application, unnecessary if the uncertainty in the dosage information is accounted for in the analysis (Jighly 2022). For a fixed amount of sequencing resources available, there is a trade-off between the number of SNPs called and the average read depth at a SNP. Results from our simulation indicate that the optimal sequencing depth increases with polyploid level, ranging from 2–3 (diploids) to 12–13 (octoploids) for relatedness and 4–5 (diploids) to 19–20 (octoploids) for self-relatedness estimates. We also found that relative to the optimal depth, slightly higher depths had a smaller impact on GRM estimates compared to slightly lower depths. These observations suggest that it is important to obtain sufficient average read depths in polyploid HTS studies, but that sequencing at an optimal low depth may still be more efficient than sequencing at very high depths. We would recommend that researchers make informed decisions based on our results as well as the ploidy of their species, availability of sequencing resources and the intended application of the GRM in their study.

There are many methods available for estimating genetic relatedness in diploid species, but suitable methods for polyploid species are limited, especially for HTS data. GUSrelate provides researchers with a tool for constructing GRMs for autopolyploid species from HTS data for use in various applications. The package accounts for errors from missing parental alleles due to low read depths and sequencing errors, which reduces bias in relatedness estimates of the GRM. This allows researchers to use HTS data more effectively and sequence at lower depths to reduce costs. We believe this tool will benefit researchers investigating autopolyploid species and consequently help advance our knowledge of polyploid species.

### Supplementary Information

Below is the link to the electronic supplementary material.

Supplementary file 1 (PDF 187 kb)

Supplementary file 2 (DOCX 1384 kb)

Supplementary file 3 (CSV 3851 kb)

Supplementary file 4 (ZIP 22612 kb)

Supplementary file 5 (CSV 7 kb)

Supplementary file 6 (ZIP 54882 kb)

## Data Availability

Scripts for performing the simulation study and summarizing the simulation results are available on GitHub at https://github.com/tpbilton/GRM_Autoploidy_HTS_data. The SNP array Potato dataset was obtained from the study by Sharma et al. (2018) and the filtered genotype matrix used in the analysis is available in Supplementary File S3. The GBS potato dataset was originally published in Sharma et al. (2023) and the counts for the reference and alternate alleles of the filtered data is available in Supplementary File S4. Supplementary File S5 is the data file for matching the IDs from the SNP array and GBS datasets and an R script for reproducing the results for the Potato dataset is available from GitHub at https://github.com/tpbilton/GRM_Autoploidy_HTS_data. Supplementary File S7 is the processed GBS data that have not been filtered for HWE.
