# Supplementary material for: Construction of relatedness matrices in autopolyploid populations using low-depth high-throughput sequencing data
Source: Theor Appl Genet. 2024 Mar 2;137(3):64. doi: 10.1007/s00122-024-04568-2 (PMC10908621; doi:10.1007/s00122-024-04568-2)
Supplement: Supplementary file 1 — (PDF 187 kb) [file 122_2024_4568_MOESM1_ESM.pdf]

Supplementary Methods: Construction of relatedness matrices in autopolyploid populations using low depth high-throughput sequencing data

Timothy P. Bilton[1,2*], Sanjeev Kumar Sharma[3], Matthew R. Schofield[2], Michael A. Black[4], Jeanne M. E. Jacobs[5], Glenn J. Bryan[3] and Ken G. Dodds[1]

[1]AgResearch,Invermay Agricultural Centre, Mosgiel, New Zealand

[2]Department of Mathematics and Statistics, University of Otago, Dunedin, New Zealand

[3]Cell and Molecular Sciences, The James Hutton Institute, Invergowrie, Dundee, United Kingdom

[4] Department of Biochemistry, University of Otago, Dunedin, New Zealand

[5]AgResearch, Lincoln Science Centre, Christchurch, New Zealand

1 Mathematical Derivations

1.1 GRM polyploid estimator for genotype data

Suppose that the alleles an individual inherits in an autopolyploid population follows a correlated binomial model as described in Equations (3)-(5). We now formalize this model. Let U_{ijmp} denote a Bernoulli random variable such that $U_{ijmp} = 1$ if the allele on the mth chromosome derived from parent p in individual i at SNP j is the reference allele and $U_{ijmp} = 0$ if the allele is the alternative allele, where $m = 1, \ldots, \omega_{ip}$, $p = 0$ for the maternal parent, $p = 1$ for the paternal parent and ω_{ip} denotes the number of alleles individual i inherits from parent p. Since

U_{ijmp} is a Bernoulli random variable, its first two moments are

$$E(U_{ijmp}) = p_j \tag{A1}$$

$$Var(U_{ijmp}) = p_j(1 - p_j). \tag{A2}$$

Since the probability that two alleles within an individual are IBD is F_i, we assume that the correlation between two of these Bernoulli variables is $Corr(U_{ijmp}, U_{ijnq}) = F_i$ for $m \neq n$, where $m = 1, \dots, \omega_{ip}$, $n = 1, \dots, \omega_{iq}$ and $p, q = 0, 1$. Under the assumption that the alleles an individual inherits from a parent are a correlated sample from the reference population alleles, $X_{ij} = \sum_{p=0}^{1}(U_{ij1p} + \dots + U_{ij\omega_{ip}p})$, where X_{ij} is now a random variable from a correlated binomial distribution. It follows that

$$E(X_{ij}) = \psi_i p_j \tag{A3}$$

$$E(X_{ij}^2) = \psi_i p_j(1 - p_j)(1 + \zeta_{ij}) + \psi_i^2 p_j^2, \tag{A4}$$

where $\psi_i = \omega_{i0} + \omega_{i1}$ and

$$\zeta_{ij} = \frac{1}{\psi_i} \sum_{p=0}^{1} \sum_{q=0}^{1} \sum_{m=1}^{\omega_p} \sum_{\substack{n=1 \\ n \neq m \Leftrightarrow p=q}}^{\omega_q} Corr(U_{ijmp}, U_{ijnq}) = (\psi_i - 1)F_i. \tag{A5}$$

The correlated binomial distribution is often used to model overdispersion and underdispersion in binomial distributions. When $\zeta_{ij} = 0$, the correlated binomial model simplifies to a standard binomial distribution.

The covariance of the genotype calls between two individuals can also be derived under the correlated binomial model from first principles. Under the correlation definition of the coancestry coefficient, the correlation of two alleles derived from different individuals is simply $Corr(U_{hjmp}, U_{ijnq}) = \theta_{hi}$. It follows that

$$\begin{aligned} Cov(X_{hj}, X_{ij}) &= \sum_{p=0}^{1} \sum_{q=0}^{1} \sum_{m=1}^{\omega_{hp}} \sum_{n=1}^{\omega_{iq}} Corr(U_{hjmp}, U_{ijnq}) \sqrt{Var(U_{hjmp}) Var(U_{ijnq})} \\ &= \psi_h \psi_i p_j (1 - p_j) \theta_{hi} \end{aligned} \tag{A6}$$

and

$$
\begin{aligned}
E(X_{hj}X_{ij}) &= Cov(X_{hj}, X_{ij}) + E(X_{hj})E(X_{ij}) \\
&= \psi_h\psi_i p_j(1-p_j)\theta_{hi} + \psi_h\psi_i p_j^2. \qquad \text{(A7)}
\end{aligned}
$$

When $h = i$, the expectation in (A7) is equivalent to (A4) since $\theta_{ii} = (1 + (\psi_i - 1)F_i)/\psi_i$.

It follows from Equations (A3),(A4) and (A7) that the expectation of the quantity $(X_{hj} - \psi_h p_j)(X_{ij} - \psi_i p_j)$ is

$$
\begin{aligned}
E\left[(X_{hj} - \psi_h p_j)(X_{ij} - \psi_i p_j)\right] &= E(X_{hj}X_{ij}) - \psi_i p_j E(X_{hj}) - \psi_h p_j E(X_{ij}) + \psi_h\psi_i p_j^2 \\
&= \psi_h\psi_i p_j(1-p_j)\theta_{hi} + \psi_h\psi_i p_j^2 - 2\psi_h\psi_i p_j^2 + \psi_h\psi_i p_j^2 \\
&= \psi_h\psi_i p_j(1-p_j)\theta_{hi} \qquad \text{(A8)}
\end{aligned}
$$

Equating this expectation to its sample quantity gives

$$
\begin{aligned}
\psi_h\psi_i p_j(1-p_j)\theta_{hi} &= (X_{hj} - \psi_h p_j)(X_{ij} - \psi_i p_j) \\
\Rightarrow \qquad \sqrt{\psi_h\psi_i}\theta_{hi} &= \frac{\sum_{j=1}^{M}(X_{hj} - \psi_h p_j)(X_{ij} - \psi_i p_j)}{\sqrt{\psi_h\psi_i}\sum_{j=1}^{M} p_j(1-p_j)} \qquad \text{(A9)}
\end{aligned}
$$

which yields the GRM estimator in Equation (6).

1.2 GRM polyploid estimator for HTS data

We make the assumption that the number of reference reads, Y_{ij}, observed in individual i at SNP j in HTS data follows the binomial model specified in Equation (9). Now, the mean of this binomial model is

$$
E(Y_{ij}|X_{ij} = x) = d_{ij}\frac{x + (\psi_i - 2x)\varepsilon_j}{\psi_i}. \qquad \text{(A10)}
$$

It follows that

$$
\begin{aligned}
E(Z_{ij}) &= \frac{\psi_i}{d_{ij}} \sum_{y=0}^{d_{ij}} y P(Y_{ij} = y) \\
&= \frac{\psi_i}{d_{ij}} \sum_{x=0}^{\psi_i} \left[\sum_{y=0}^{d_{ij}} y P(Y_{ij} = y | X_{ij} = x) \right] P(X_{ij} = x) \\
&= \frac{\psi_i}{d_{ij}} \sum_{x=0}^{\psi_i} d_{ij} \left(\frac{x + (\psi_i - 2x)\varepsilon_j}{\psi_i} \right) P(X_{ij} = x) \\
&= \psi_i \varepsilon_j + (1 - 2\varepsilon_j) E(X_{ij}) \qquad \text{(A11)}
\end{aligned}
$$

which gives Equation (11), where $Z_{ij} = \psi_i Y_{ij}/d_{ij}$. Furthermore, the variance of the binomial model in Equation (9) is

$$
\begin{aligned}
Var(Y_{ij} | X_{ij} = x) &= d_{ij} \left(\frac{x + (\psi_i - 2x)\varepsilon_j}{\psi_i} \right) \left(1 - \frac{x + (\psi_i - 2x)\varepsilon_j}{\psi_i} \right) \\
&= \frac{d_{ij}}{\psi_i^2} (\psi_i^2 \varepsilon_j (1 - \varepsilon_j) + x(\psi_i - x)\gamma_j) \qquad \text{(A12)}
\end{aligned}
$$

where $\gamma_j = 1 - 4\varepsilon_j(1 - \varepsilon_j)$. Furthermore,

$$
[E(Y_{ij} | X_{ij} = x)]^2 = \frac{d_{ij}^2}{\psi_i^2} (\psi_i^2 \varepsilon_j^2 + 2\psi_i x (1 - 2\varepsilon_j) + \gamma_j x^2) \qquad \text{(A13)}
$$

It follows from (A12) and (A13) that

$$
E(Y_{ij}^2 | X_{ij} = x) = \frac{d_{ij}^2}{\psi_i^2} \left[\psi_i^2 (d_{ij} \varepsilon_j^2 + \varepsilon_j (1 - \varepsilon_j)) + \psi_i (2 d_{ij} (1 - 2\varepsilon_j) + \gamma_j)) x + (d_{ij} - 1)\gamma_j x^2 \right] \qquad \text{(A14)}
$$

and that

$$
\begin{aligned}
E(Z_{ij}^2) &= \frac{\psi_i^2}{d_{ij}^2} \sum_{y=0}^{d_{ij}} y^2 P(Y_{ij} = y) \\
&= \frac{\psi_i^2}{d_{ij}^2} \sum_{x=0}^{\psi_i} \left[\sum_{y=0}^{d_{ij}} y^2 P(Y_{ij} = y | X_{ij} = x) \right] P(X_{ij} = x) \\
&= \frac{\psi_i^2}{d_{ij}^2} \sum_{x=0}^{\psi_i} E(Y_{ij}^2 | X_{ij} = x) P(X_{ij} = x) \\
&= \psi_i^2 \varepsilon_j (1 - (1 - \varepsilon_j)\delta_{ij}) + \psi_i \left(1 + 2\varepsilon_j(1 - 2\varepsilon_j) - \gamma_j \delta_{ij}\right) E(X_{ij}) + \gamma_j \delta_{ij} E(X_{ij}^2)
\end{aligned} \quad \text{(A15)}
$$

which yields Equation (12), where $\delta_{ij} = 1 - 1/d_{ij}$. It also follows from Equation (A10) that

$$
\begin{aligned}
E(Z_{hj}, Z_{ij}) &= \frac{\psi_h \psi_i}{d_{hj} d_{ij}} \sum_{y_h=0}^{d_{hj}} \sum_{y_i=0}^{d_{ij}} y_h y_i P(Y_{hj} = y_h, Y_{ij} = y_i) \\
&= \frac{\psi_h \psi_i}{d_{hj} d_{ij}} \sum_{x_h=0}^{\psi_h} \sum_{x_i=0}^{\psi_i} \left[\sum_{y_h=0}^{d_{hj}} y_h P(Y_{hj} = y_h | X_{hj} = x_h) \right] \left[\sum_{y_i=0}^{d_{ij}} y_i P(Y_{ij} = y_i | X_{ij} = x_i) \right] \times \\
&\qquad P(X_{hj} = x_h | X_{ij} = x_i) \\
&= \sum_{x_h=0}^{\psi_h} \sum_{x_i=0}^{\psi_i} \left(x_h + (\psi_h - 2x_h)\varepsilon_j\right) \left(x_i + (\psi_i - 2x_i)\varepsilon_j\right) \\
&= \gamma_j E(X_{hj} X_{ij}) + \varepsilon_j (1 - 2\varepsilon_j)(\psi_i E(X_{hj}) + \psi_h E(X_{ij})) + \psi_h \psi_i \varepsilon_j^2
\end{aligned} \quad \text{(A16)}
$$

which yields Equation (13). From Equations (A11), (A15) and (A16), the expectations of the quantity $(Z_{hj} - \psi_h p_j)(Z_{ij} - \psi_i p_j)$ are

$$
\begin{aligned}
E((Z_{ij} - \psi_i p_j)^2) &= E(Z_{ij}) - 2\psi_i p_j E(Z_{ij}) + \psi_i^2 p_j^2 \\
&= \gamma_j \delta_{ij} \psi_i^2 p_j (1 - p_j)(1 + (\psi_i - 1)F_i) + \psi_i^2 p_j (1 - p_j)(1 - \gamma_j \delta_{ij}) + \\
&\qquad \psi_i^2 \varepsilon_j \left(1 - (1 - \varepsilon_j)\delta_{ij} - 4p_j(1 - p_j)\right)
\end{aligned} \quad \text{(A17)}
$$

for the diagonal elements ($h = i$), and

$$\begin{aligned}E((Z_{hj}-\psi_h p_j)(Z_{ij}-\psi_i p_j)) &= E(Z_{hj}Z_{ij}) - p_j(\psi_i E(Z_{hj}) + \psi_h E(Z_{ij})) + \psi_h\psi_i p_j^2 \\ &= \gamma_j\psi_h\psi_i(p_j(1-p_j)\theta_{hi} + p_j^2) + 2\varepsilon_j(1-2\varepsilon_j)\psi_h\psi_i p_j + \psi_h\psi_i\varepsilon_j^2 - \\ &\quad 2\psi_h\psi_i p_j(\varepsilon_j + (1-2\varepsilon_j)p_j) + \psi_h\psi_i p_j^2 \\ &= \gamma_j\psi_h\psi_i p_j(1-p_j)\theta_{hi} + \psi_h\psi_i\varepsilon_j^2\eta_j \end{aligned} \tag{A18}$$

for the off-diagonal elements ($h \neq i$), where $\eta_j = 1 - 4p_j(1-p_j)$. Equating the expectation in Equation (A17) to its sample quantity gives

$$1 + (\psi_i - 1)F_i = \frac{((Z_{ij} - \psi_i p_j)^2 - \psi_i^2 A_{ij})/(\gamma_j\delta_{ij})}{\psi_i p_j(1-p_i)}, \qquad d_{ij} > 1 \tag{A19}$$

where $A_{ij} = (p_j(1-p_j)(1-\gamma_j\delta_{ij}) + \varepsilon_j(\eta_j - (1-\varepsilon_j)\delta_{ij})$. Taking the summation over the SNPs in both the numerator and denominator in Equation (A19) and rearranging slightly results in the unbiased GRM estimator given in Equation (15). In addition, equating the expectation in Equation (A18) to its sample quantity gives

$$\sqrt{\psi_h\psi_i}\theta_{hi} = \frac{((Z_{hj} - \psi_h p_j)(Z_{ij} - \psi_i p_j) - \psi_h\psi_i\varepsilon_j^2(1 - 4p_j(1-p_j)))/\gamma_j}{\sqrt{\psi_h\psi_i}p_j(1-p_j)}, \qquad h \neq i \tag{A20}$$

which, after summing over the SNPs in both the numerator and denominator, and rearranging slightly results in the unbiased GRM estimator given in Equation (14).

1.3 Missing data

Missing data occur in HTS data when there are no reads at a given SNP position for an individual. The estimators derived in Equation (A9) for genotype data and Equations (A19) and (A20) for HTS data both assume complete genotype information. To handle missing data when constructing these estimators, we utilize the approach by Dodds *et al.* (2015) whereby missingness is assumed at random and the GRM is constructed for a pair of individuals using only the SNPs with sufficient information in both individuals via an efficient algorithm based on matrix

computation. We now outline this approach.

Define the matrices

$$(\mathbf{R}_d)_{ij} = \left(\begin{cases} r_{ij} & d_{ij} \geq d \\ 0 & \text{otherwise} \end{cases}\right) \tag{A21}$$

as the $N \times M$ matrix of observed read count ratios (where r_{ij} is the realized value of $R_{ij} = Y_{ij}/d_{ij}$),

$$(\mathbf{D}_d)_{ij} = \left(\begin{cases} \frac{1}{d_{ij}} & d_{ij} \geq d \\ 0 & \text{otherwise} \end{cases}\right) \tag{A22}$$

as the $N \times M$ matrix of observed read depths,

$$(\mathbf{P}_d)_{ij} = \left(\begin{cases} p_j & d_{ij} \geq d \\ 0 & \text{otherwise} \end{cases}\right) \tag{A23}$$

as the $N \times M$ matrix of allele frequencies,

$$(\mathbf{E}_d)_{ij} = \left(\begin{cases} \varepsilon_j & d_{ij} \geq d \\ 0 & \text{otherwise} \end{cases}\right) \tag{A24}$$

as the $N \times M$ matrix of sequencing errors, and

$$(\mathbf{\Psi})_{hi} = \left(\begin{cases} \psi_i & h = i \\ \sqrt{\psi_h \psi_i} & h \neq i \end{cases}\right) \tag{A25}$$

as the $N \times N$ matrix of average ploidy levels between each pair of individuals, where the notation $(\boldsymbol{A})_{rc}$ defines the element in the rth row and c^{th} column for the matrix $\boldsymbol{A}$. The matrices defined in (A21)-(A24) are defined so that missing data has zero contribution to the numerator and

denominator when computing the GRM using these matrices. Define the matrices

$$\mathbf{Q}_1 = (\mathbf{R}_1 - \mathbf{P}_1) \oslash ((1 - 4\mathbf{E}_1 \odot (1 - \mathbf{E}_1))^{\odot 0.5}) \tag{A26}$$

$$\mathbf{Q}_2 = (\mathbf{E}_1 \odot \mathbf{E}_1 \odot (1 - 4\mathbf{P}_1 \odot (1 - \mathbf{P}_1)))^{\odot 0.5} \oslash ((1 - 4\mathbf{E}_1 \odot (1 - \mathbf{E}_1))^{\odot 0.5}) \tag{A27}$$

$$\begin{aligned}\mathbf{Q}_3 = [&\mathbf{P}_2 \odot (1 - \mathbf{P}_2) \odot (\mathbf{D}_2 + 4\mathbf{E}_2 \odot (1 - \mathbf{E}_2) \odot (1 - \mathbf{D}_2) + \\ &\mathbf{E}_2 \odot (\mathbf{E}_2 \odot \mathbf{D}_2 \odot (1 - \mathbf{E}_2) + \mathbf{D}_2 - 4\mathbf{P}_2 \odot (1 - \mathbf{P}_2)] \oslash (1 - 4\mathbf{E}_1 \odot (1 - \mathbf{E}_1)),\end{aligned} \tag{A28}$$

where $\odot$ denotes the Hadamard product (i.e., componentwise matrix multiplication), $\mathbf{M}^{\odot n}$ denotes the Hadamard power of n (i.e., taking the power of n for each element of a matrix) and $\oslash$ denotes the Hadamard division (i.e., componentwise matrix division). The estimator defined in (A19) and (A20) can be computed with missing data by first computing the matrix

$$\boldsymbol{G} = \boldsymbol{\Psi} \odot (\mathbf{Q}_1^T \mathbf{Q}_1 - \mathbf{Q}_2^T \mathbf{Q}_2) \oslash (\mathbf{P}_1^T (1 - \mathbf{P}_1)) \tag{A29}$$

and then recomputing the diagonal elements using

$$\mathrm{diag}(\boldsymbol{G}) = \mathrm{diag}(\boldsymbol{\Psi}) \odot (((\mathbf{R}_2 - \mathbf{P}_2)^{\odot 2} - \mathbf{Q}_3)\mathbf{1}_M^T) \oslash (\mathbf{P}_2 \odot (1 - \mathbf{P}_2)\mathbf{1}_M^T), \tag{A30}$$

where $\mathbf{1}_M$ denotes a row vector of 1's with length M, diag($\mathbf{Q}$) denotes the diagonal elements of a matrix $\mathbf{Q}$, and $\boldsymbol{A}^T$ denotes the transpose of the matrix $\boldsymbol{A}$.

References

Dodds, K. G., J. C. McEwan, R. Brauning, R. M. Anderson, T. C. Van Stijn, *et al.*, 2015 Construction of relatedness matrices using genotyping-by-sequencing data. BMC Genomics **16**: 1047.
