# Supplementary material for: Construction of relatedness matrices in autopolyploid populations using low-depth high-throughput sequencing data
Source: Theor Appl Genet. 2024 Mar 2;137(3):64. doi: 10.1007/s00122-024-04568-2 (PMC10908621; doi:10.1007/s00122-024-04568-2)
Supplement: Supplementary file 2 — (DOCX 1384 kb) [file 122_2024_4568_MOESM2_ESM.docx]

**Construction of relatedness matrices in autopolyploid populations using low-depth high-throughput sequencing data**

Timothy P. Bilton^1,2*^, Sanjeev Kumar Sharma^3^, Matthew R. Schofield^2^, Michael A. Black^4^, Jeanne M. E. Jacobs^5^, Glenn J. Bryan^3^, Ken G. Dodds^1^

^1^Invermay Agricultural Centre, AgResearch, Mosgiel, New Zealand

^2^Department of Mathematics and Statistics, University of Otago, Dunedin, New Zealand

^3^Cell and Molecular Sciences, The James Hutton Institute, Invergowrie, Dundee, United Kingdom

^4^Department of Biochemistry, University of Otago, Dunedin, New Zealand

^5^Lincoln Science Centre, AgResearch, Christchurch, New Zealand

Table S1: Parameters values varied in the second simulation set.

| Average read depth ($\mu_{d_{j}}$) | Number of SNPs ($M$) | |
| --- | --- | --- |
|  | Sequencing effort = 10M | Sequencing effort = 40M |
| 1 | 23,810 | 95,238 |
| 2 | 11,905 | 47,619 |
| 3 | 7,937 | 31,746 |
| 4 | 5,952 | 23,810 |
| 5 | 4,762 | 19,048 |
| 6 | 3,968 | 15,873 |
| 7 | 3,401 | 13,605 |
| 8 | 2,976 | 11,905 |
| 9 | 2,646 | 10,582 |
| 10 | 2,381 | 9,524 |
| 11 | 2,165 | 8,658 |
| 12 | 1,984 | 7,937 |
| 13 | 1,832 | 7,326 |
| 14 | 1,701 | 6,803 |
| 15 | 1,587 | 6,349 |
| 16 | 1,488 | 5,952 |
| 17 | 1,401 | 5,602 |
| 18 | 1,323 | 5,291 |
| 19 | 1,253 | 5,013 |
| 20 | 1,190 | 4,762 |
| 21 | 1,134 | 4,535 |
| 22 | 1,082 | 4,329 |
| 23 | 1,035 | 4,141 |
| 24 | 992 | 3,968 |
| 25 | 952 | 3,810 |
| 26 | 916 | 3,663 |
| 27 | 882 | 3,527 |
| 28 | 850 | 3,401 |
| 29 | 821 | 3,284 |
| 30 | 794 | 3,175 |
| 35 | 680 | 2,721 |
| 40 | 595 | 2,381 |


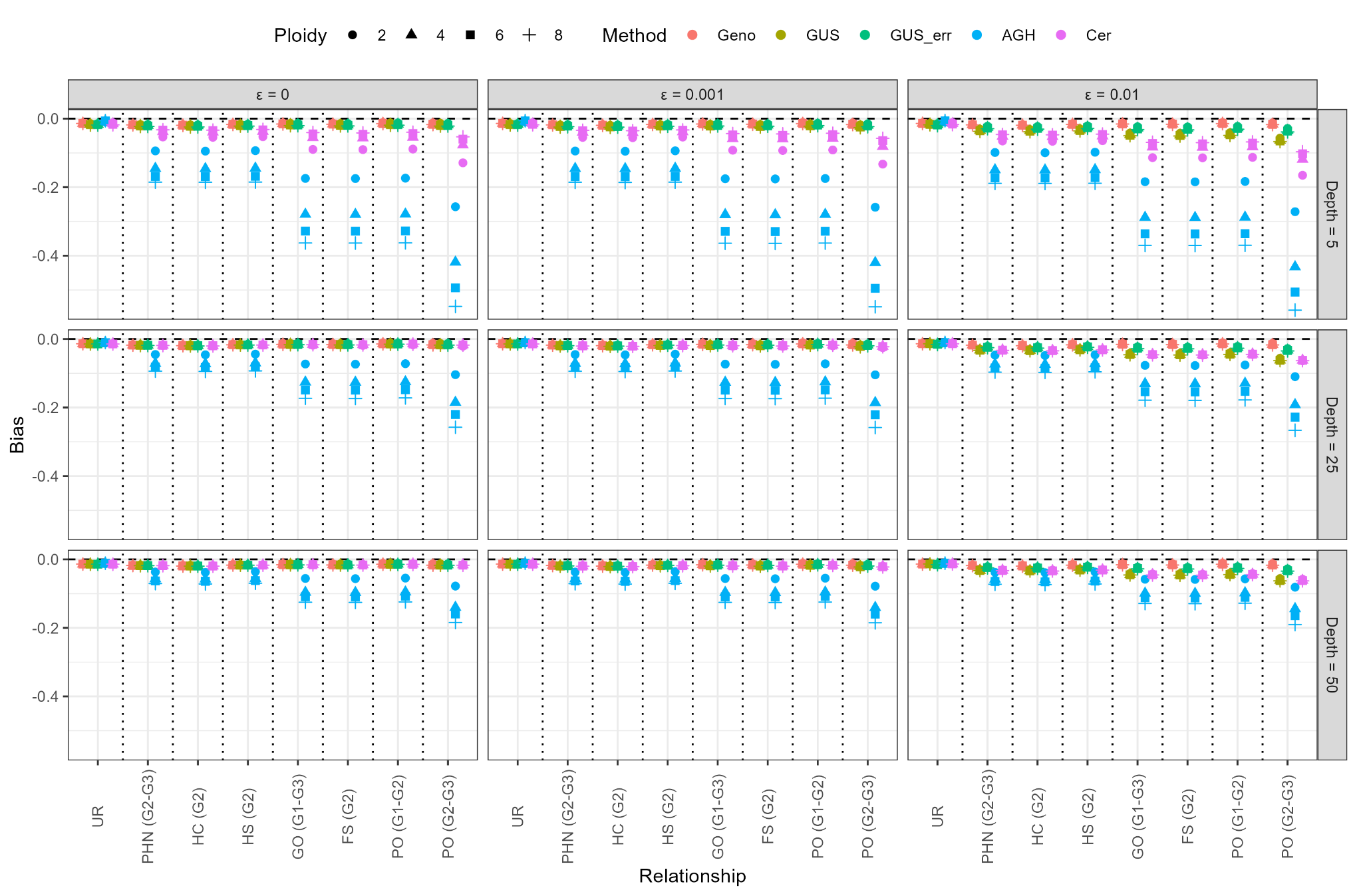


Figure S1: Bias of relatedness (off-diagonal) estimates for different GRMs. Each point represents the average bias across 500 datasets simulated using the specific combination of sequencing error, mean read depth and ploidy level, and for which pairwise relationship as specified in Table 1. Rows represent datasets simulated with average read depth of 5 (top row), 25 (middle row) and 50 (bottom row), columns represent datasets simulated with no sequencing error (first column) and a mean sequencing error of 0.1% (middle column) or 1% (right column). Points are coloured based on method used to construct the GRM, where the methods used were GUSrelate with no sequencing error (GUS) and using the true sequencing error rate (GUS_err), AGHmatrix (AGH) and Cericola et al. (2018) (Cer), which are compared to estimates from the GRM constructed using the true genotypes (Geno). The x-axis denotes whether the relationship between individuals was unrelated (UR), pibling-half nibling for Gen2 to Gen3 (PHN (G2-G3)), half cousins for Gen3 (HC (G3)), half siblings for Gen2 (HS (G2)), grandparent-offspring for Gen1 to Gen3 (GO (G1-G3)), full sibling in Gen 2 (FS (G2)), Gen1 to Gen2 (PO (G1-G2)), or parent-offspring for Gen2 to Gen3 (PO (G2-G3)), and different symbols are used to denote whether the ploidy was diploid (circle), tetraploid (triangle), hexaploid (square) or octoploid (cross) species.


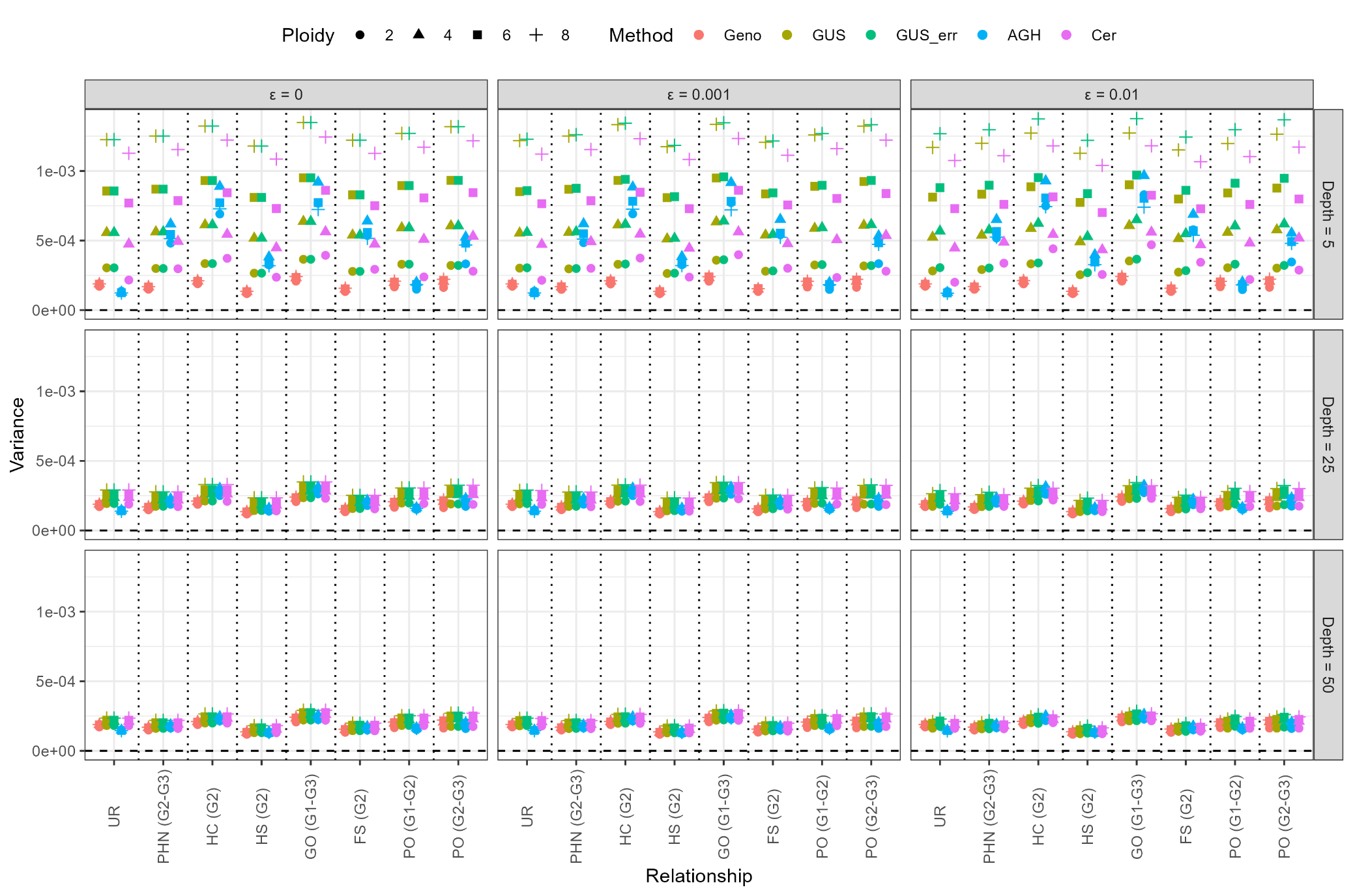


Figure S2: Variance of relatedness (off-diagonal) estimates for different GRMs. Each point represents the average variance across 500 datasets simulated using the specific combination of sequencing error, mean read depth and ploidy level, and for which pairwise relationship as specified in Table 1. Rows represent datasets simulated with average read depth of 5 (top row), 25 (middle row) and 50 (bottom row), columns represent datasets simulated with no sequencing error (first column) and a mean sequencing error of 0.1% (middle column) or 1% (right column). Points are coloured based on method used to construct the GRM, where the methods used were GUSrelate with no sequencing error (GUS) and using the true sequencing error rate (GUS_err), AGHmatrix (AGH) and Cericola et al. (2018) (Cer), which are compared to estimates from the GRM constructed using the true genotypes (Geno). The x-axis denotes whether the relationship between individuals was unrelated (UR), pibling-half nibling for Gen2 to Gen3 (PHN (G2-G3)), half cousins for Gen3 (HC (G3)), half siblings for Gen2 (HS (G2)), grandparent-offspring for Gen1 to Gen3 (GO (G1-G3)), full sibling in Gen 2 (FS (G2)), Gen1 to Gen2 (PO (G1-G2)), or parent-offspring for Gen2 to Gen3 (PO (G2-G3)), and different symbols are used to denote whether the ploidy was diploid (circle), tetraploid (triangle), hexaploid (square) or octoploid (cross) species.


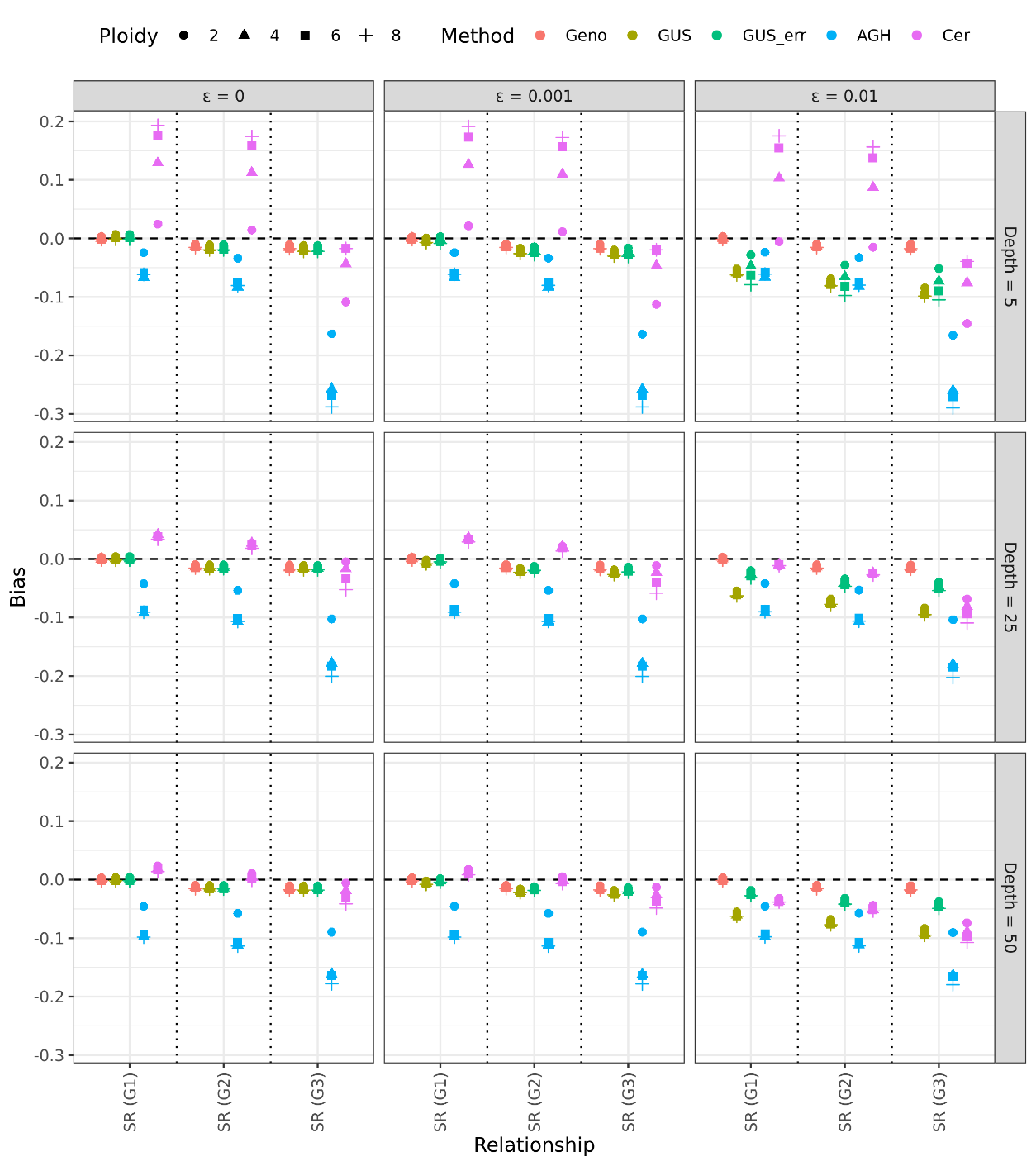


Figure S3: Bias of self-relatedness (diagonal) estimates for different GRMs. Each point represents the average bias across 500 datasets simulated using the specific combination of sequencing error, mean read depth and ploidy level, and for different generations. Rows represent datasets simulated with average read depth of 5 (top row), 25 (middle row) and 50 (bottom row), columns represent datasets simulated with no sequencing error (first column), and a mean sequencing error of 0.1% (middle column) or 1% (right column). Points are coloured based on method used to construct the GRM, where the methods used were GUSrelate with no sequencing error (GUS) and using the true sequencing error rate (GUS_err), AGHmatrix (AGH) and Cericola et al. (2018) (Cer), which are compared to estimates from the GRM constructed using the true genotypes (Geno). The x-axis denotes whether the relationship between individuals was for individuals in generation 1 (SR (G1)), generation 2 (SR (G2)) or generation 3 (SR (G3)), and different symbols are used to denote whether the ploidy was diploid (circle), tetraploid (triangle), hexaploid (square) or octoploid (cross) species.


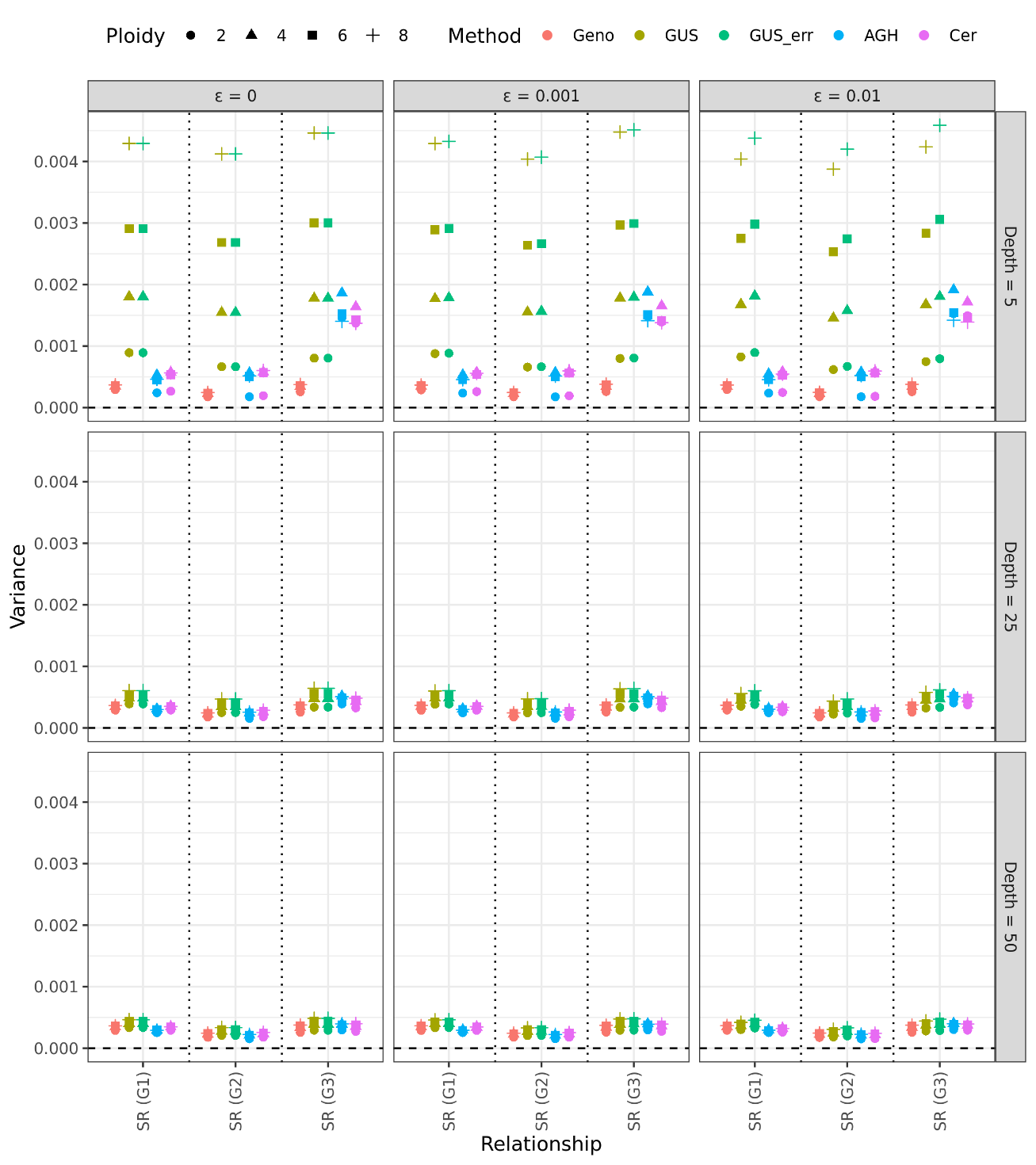


Figure S4: Variance of self-relatedness (diagonal) estimates for different GRMs. Each point represents the average variance across 500 datasets simulated using the specific combination of sequencing error, mean read depth and ploidy level, and for different generations. Rows represent datasets simulated with average read depth of 5 (top row), 25 (middle row) and 50 (bottom row), columns represent datasets simulated with no sequencing error (first column), and a mean sequencing error of 0.1% (middle column) or 1% (right column). Points are coloured based on method used to construct the GRM, where the methods used were GUSrelate with no sequencing error (GUS) and using the true sequencing error rate (GUS_err), AGHmatrix (AGH) and Cericola et al. (2018) (Cer), which are compared to estimates from the GRM constructed using the true genotypes (Geno). The x-axis denotes whether the relationship between individuals was for individuals in generation 1 (SR (G1)), generation 2 (SR (G2)) or generation 3 (SR (G3)), and different symbols are used to denote whether the ploidy was diploid (circle), tetraploid (triangle), hexaploid (square) or octoploid (cross) species.


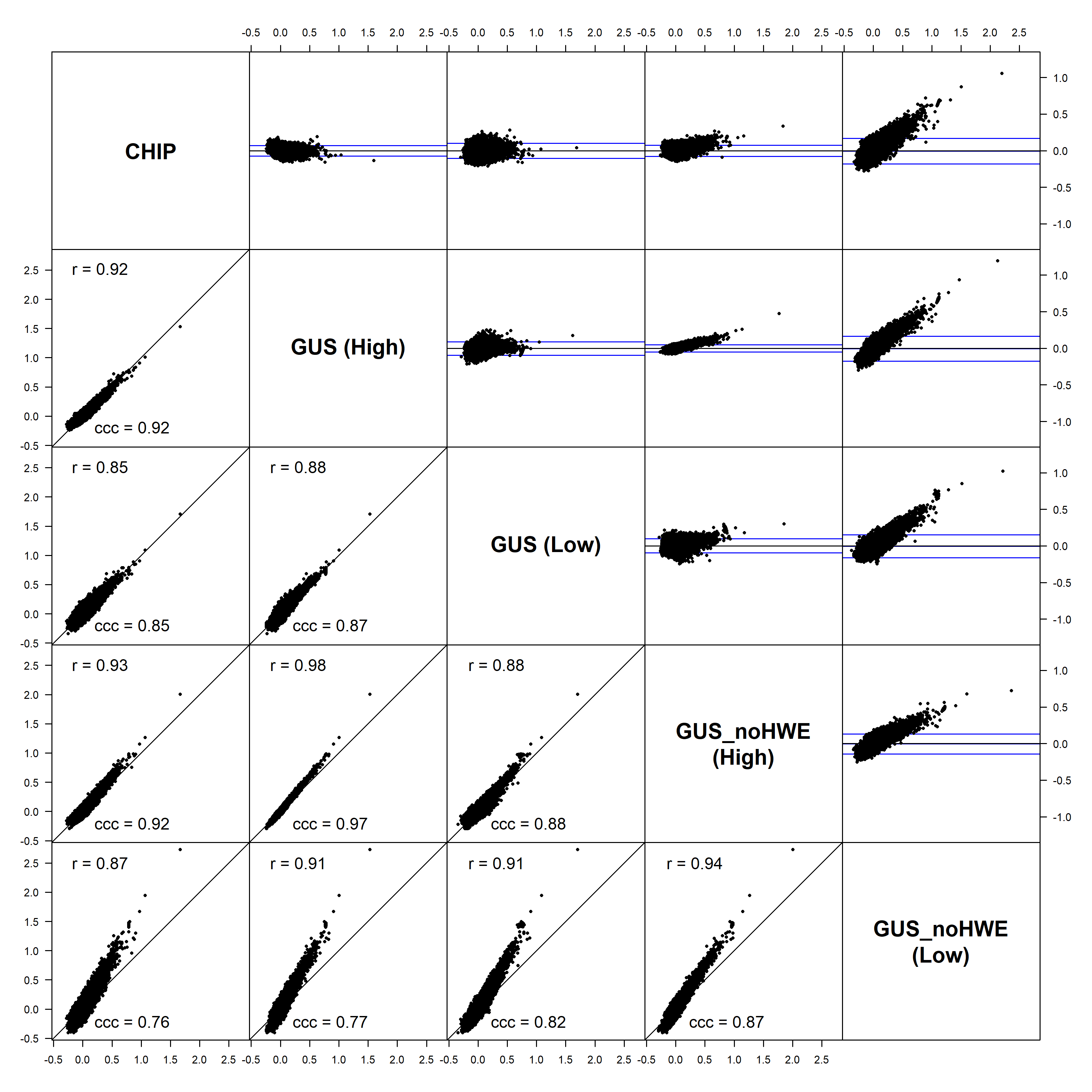


Figure S5: Matrix plot comparing the relatedness (off-diagonal) estimates between different GRMs. Lower diagonals show scatter plots of self-relatedness estimates between two GRMs along with spearman's correlation coefficient (r) and Lin’s concordance correlation coefficient (ccc) for each pair of GRMs and the upper diagonals show the corresponding Bland-Altman plot. The combination of dataset and method used to construct the GRM is specified on the diagonal, and correspond to the codes given in Cericola et al. (2018), except that for “GUS_noHWE (High)” which is the GRM constructed using high depth SNPs with no HWE filter (59243 SNPs in total) and “GUS_noHWE (Low)” which is the GRM constructed using low depth SNPs with no HWE filter (60243 SNPs in total).


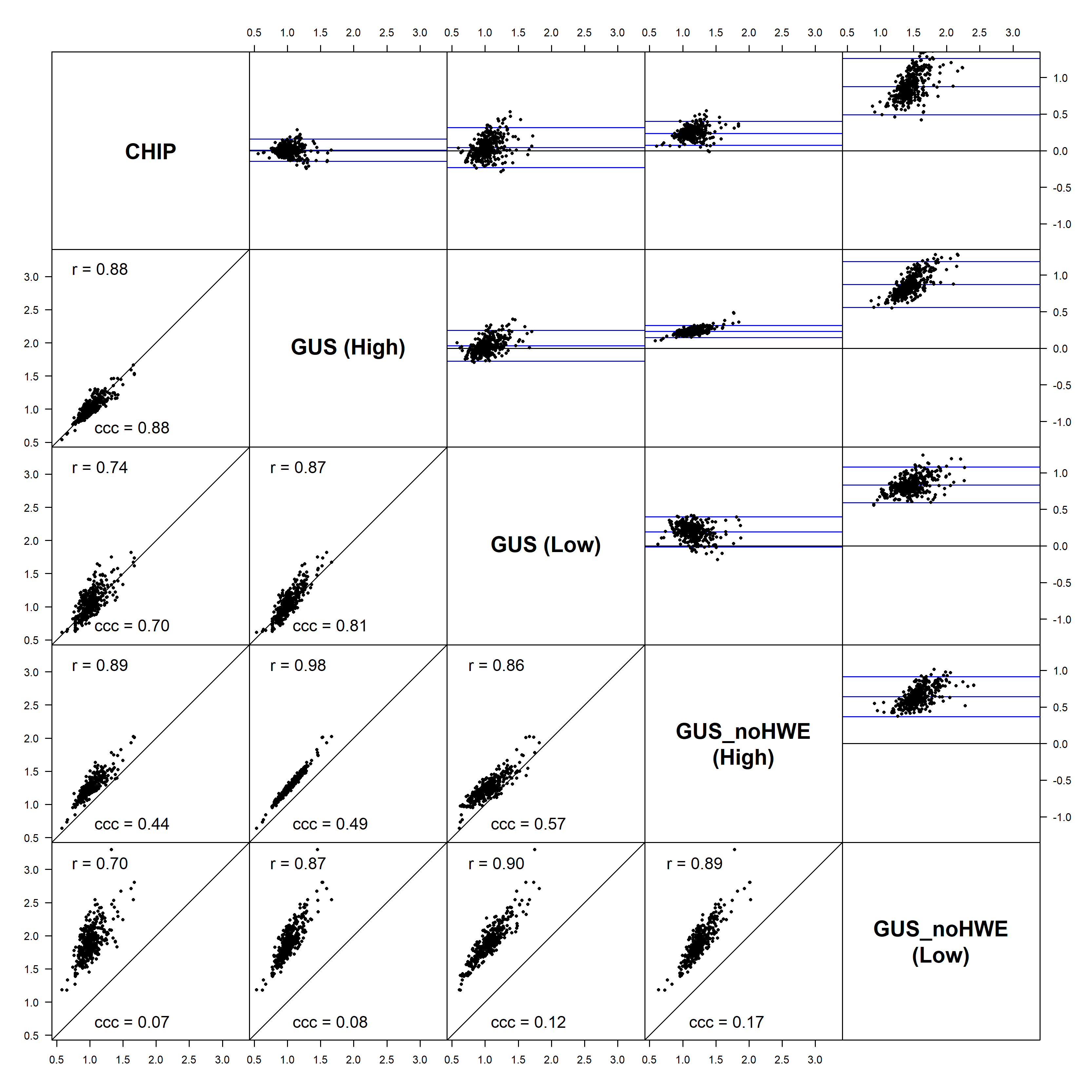


Figure S6: Matrix plot comparing the self-relatedness (diagonal) estimates between different GRMs. Lower diagonals show scatter plots of self-relatedness estimates between two GRMs along with spearman's correlation coefficient (r) and Lin’s concordance correlation coefficient (ccc) for each pair of GRMs and the upper diagonals show the corresponding Bland-Altman plot. The combination of dataset and method used to construct the GRM is specified on the diagonal, and correspond to the codes given in Cericola et al. (2018), except that for “GUS_noHWE (High)” which is the GRM constructed using high depth SNPs with no HWE filter (59243 SNPs in total) and “GUS_noHWE (Low)” which is the GRM constructed using low depth SNPs with no HWE filter (60243 SNPs in total).

**References:**

Cericola F, Lenk I, Fè D, Byrne S, Jensen CS, Pedersen MG, Asp T, Jensen J, Janss L (2018) Optimized use of low-depth genotyping-by-sequencing for genomic prediction among multi-parental family pools and single plants in perennial ryegrass (*Lolium perenne* L.). Front Plant Sci 9:369
